# An integrated deep learning model for the prediction of pathological complete response to neoadjuvant chemotherapy with serial ultrasonography in breast cancer patients: a multicentre, retrospective study

**DOI:** 10.1186/s13058-022-01580-6

**Published:** 2022-11-21

**Authors:** Lei Wu, Weitao Ye, Yu Liu, Dong Chen, Yuxiang Wang, Yanfen Cui, Zhenhui Li, Pinxiong Li, Zhen Li, Zaiyi Liu, Min Liu, Changhong Liang, Xiaotang Yang, Yu Xie, Ying Wang

**Affiliations:** 1 Department of Radiology, Guangdong Provincial People’s Hospital, Guangdong Academy of Medical Sciences, 106 Zhongshan 2nd Road, Guangzhou, 510080 China; 2 Guangdong Provincial Key Laboratory of Artificial Intelligence in Medical Image Analysis and Application, Guangdong Provincial People’s Hospital, Guangdong Academy of Medical Sciences, Guangzhou, 510080 China; 3 Guangdong Cardiovascular Institute, 106 Zhongshan 2nd Road, Guangzhou, 510080 China; 4 Department of Ultrasound, Guangdong Provincial People’s Hospital, Guangdong Academy of Medical Sciences, 106 Zhongshan 2nd Road, Guangzhou, 510080 China; 5 Department of Medical Ultrasound, Yunnan Cancer Hospital, Yunnan Cancer Center, The Third Affiliated Hospital of Kunming Medical University, Kunming, 650118 China; 6 Shanxi Province Cancer Hospital/Shanxi Hospital Affiliated to Cancer Hospital, Chinese Academy of Medical Sciences/Cancer Hospital Affiliated to Shanxi Medical University, Taiyuan, 030013 China; 7 Department of Radiology, Yunnan Cancer Hospital, Yunnan Cancer Center, The Third Affiliated Hospital of Kunming Medical University, Kunming, 650118 China; 8 Department of 3rd Breast Surgery, Yunnan Cancer Hospital, Yunnan Cancer Center, The Third Affiliated Hospital of Kunming Medical University, Kunming, 650118 China; 9 Department of Ultrasound, State Key Laboratory of Oncology in South China, Collaborative Innovation Center for Cancer Medicine, Sun Yat-Sen University Cancer Center, Guangzhou, 510060 China; 10 Department of Medical Ultrasonics, The First Affiliated Hospital of Guangzhou Medical University, 151 Yanjiang West Road, Guangzhou, 510120 China

**Keywords:** Deep learning, Breast cancer, Neoadjuvant chemotherapy, Serial ultrasonography

## Abstract

**Background:**

The biological phenotype of tumours evolves during neoadjuvant chemotherapy (NAC). Accurate prediction of pathological complete response (pCR) to NAC in the early-stage or posttreatment can optimize treatment strategies or improve the breast-conserving rate. This study aimed to develop and validate an autosegmentation-based serial ultrasonography assessment system (SUAS) that incorporated serial ultrasonographic features throughout the NAC of breast cancer to predict pCR.

**Methods:**

A total of 801 patients with biopsy-proven breast cancer were retrospectively enrolled from three institutions and were split into a training cohort (242 patients), an internal validation cohort (197 patients), and two external test cohorts (212 and 150 patients). Three imaging signatures were constructed from the serial ultrasonographic features before (pretreatment signature), during the first–second cycle of (early-stage treatment signature), and after (posttreatment signature) NAC based on autosegmentation by U-net. The SUAS was constructed by subsequently integrating the pre, early-stage, and posttreatment signatures, and the incremental performance was analysed.

**Results:**

The SUAS yielded a favourable performance in predicting pCR, with areas under the receiver operating characteristic curve (AUCs) of 0.927 [95% confidence interval (CI) 0.891–0.963] and 0.914 (95% CI 0.853–0.976), compared with those of the clinicopathological prediction model [0.734 (95% CI 0.665–0.804) and 0.610 (95% CI 0.504–0.716)], and radiologist interpretation [0.632 (95% CI 0.570–0.693) and 0.724 (95% CI 0.644–0.804)] in the external test cohorts. Furthermore, similar results were also observed in the early-stage treatment of NAC [AUC 0.874 (0.793–0.955)–0.897 (0.851–0.943) in the external test cohorts].

**Conclusions:**

We demonstrate that autosegmentation-based SAUS integrating serial ultrasonographic features throughout NAC can predict pCR with favourable performance, which can facilitate individualized treatment strategies.

**Supplementary Information:**

The online version contains supplementary material available at 10.1186/s13058-022-01580-6.

## Background

Breast cancer is the most common cancer and the leading cause of cancer-related deaths in women [1]. Neoadjuvant chemotherapy (NAC) is the standard of care for patients with locally advanced breast cancer, and it is increasingly used for patients with operable breast cancer to allow more conservative surgery in the breast and axilla [2]. However, not all patients benefit from NAC, and the reported rates of pathological complete response (pCR) are generally less than 70% [3–5]. Accurate prediction of pCR allows for early intervention for non-pCR patients to increase pCR rates [6] and guides clinicians in choosing breast-conserving surgery. However, no reliable biomarkers currently exist to aid in pCR prediction.

Magnetic resonance imaging (MRI) is one of the main imaging methods used to monitor the response to NAC in breast cancer [7–9]. However, few patients can use MRI to monitor treatment response in each cycle of NAC because of its high cost and inflexibility. Ultrasound is widely used to evaluate treatment response in clinical practice due to its low cost and convenience. In addition, ultrasound is recommended by guidelines to evaluate or re-evaluate the lesions before, during, and after NAC [2, 10]. However, the performance of conventional ultrasound remains far from satisfactory, with a false negative rate (FNR) for pCR up to 39.2% [11].

Recently, radiomics has been used for breast cancer diagnosis, treatment assessment, and prognosis prediction [12–17]. Indeed, radiomics based on the analysis of medical images showed the ability to noninvasively describe tumour phenotypes with more predictive power than routine clinical methods [12]. However, in traditional quantitative image analysis, tumour segmentation is delineated manually by radiologists, which is time-consuming and has inter/intraobserver variability [12, 17]. In contrast, deep learning has certain advantages in segmentation speed and reducing variability. Nevertheless, most previous studies have focused on identifying imaging biomarkers at a single time point [16, 18, 19]. Biological behaviour is a dynamic ecosystem with various cellular contributions; hence, tumour heterogeneity may not be fully captured at a single time point [20, 21]. It may be beneficial to integrate serial ultrasound images during NAC as a way to monitor changes in tumour biological characteristics [12, 17, 22].

Thus, in this study, we developed and validated a deep learning-based serial ultrasonography assessment system (SUAS) for predicting the neoadjuvant chemotherapy response of breast cancer using serial ultrasound images.

## Methods

### Participants and data acquisition

The ethics committees of the Guangdong Provincial People’s Hospital (GPPH), Yunnan Cancer Hospital (YNCH), and Shanxi Province Cancer Hospital (SPCH) approved this multicentre retrospective study. The board waived the requirement for informed consent because of the study’s retrospective nature. All data in the study were deidentified and anonymized.

**Fig. 1:**
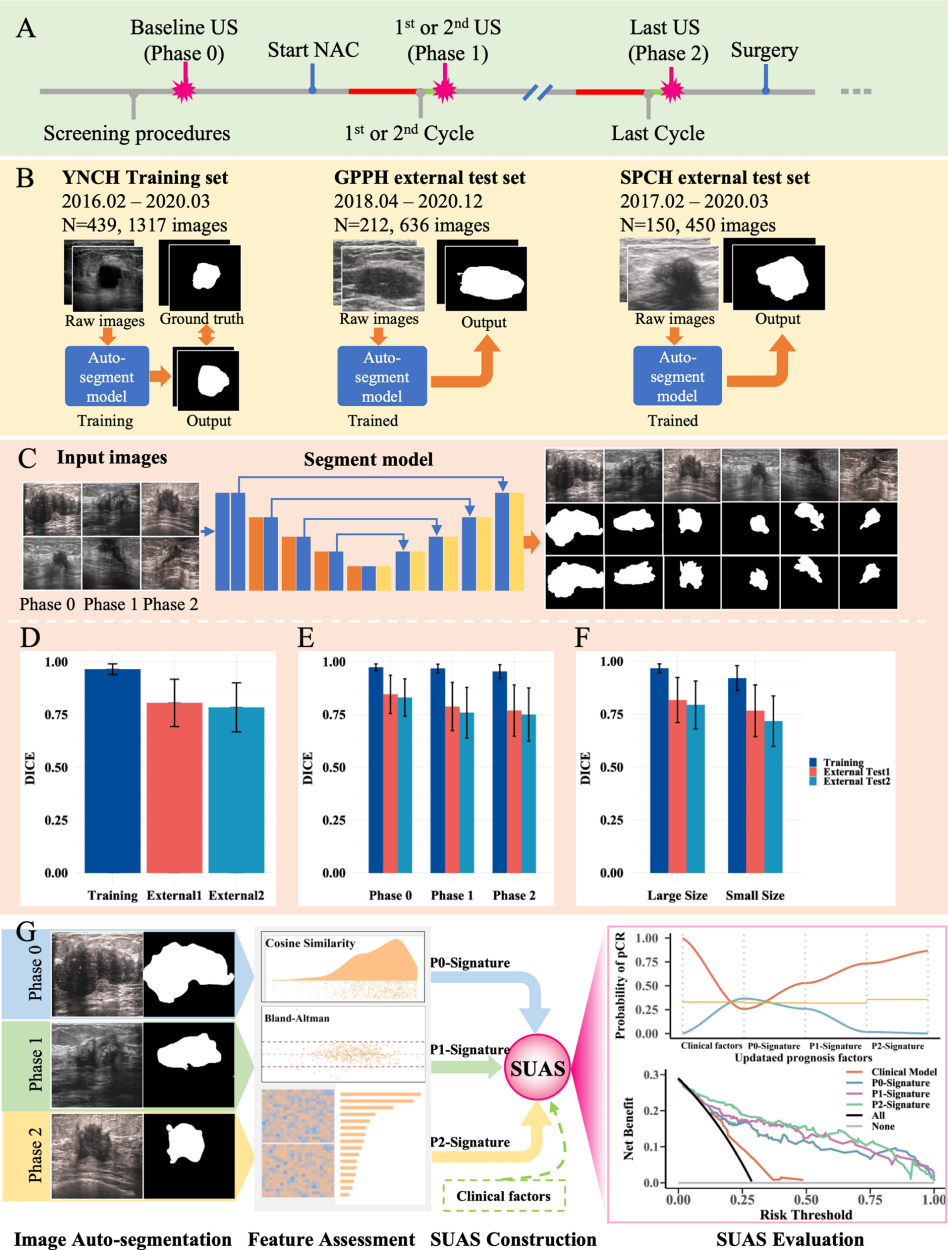
Study design and workflow. **A** Schematic diagram of ultrasound images acquisition during diagnosis and treatment of patients with breast cancer who received NAC. Pre-treatment ultrasonography (baseline ultrasonography, denoted as Phase 0), during the first–second cycle of NAC (Phase 1) and posttreatment (Phase 2) ultrasonography were acquired for each patient. **B** Patients with breast cancer undergoing NAC were divided into the training cohort (YNCH, *N* = 439, 1317 images) and two external test cohorts (GPPH, *N* = 212, 636 images, and SPCH, *N* = 150, 450 images, respectively). The YNCH cohort was used for training an automated tumour segmentation model, and the performance was tested in the GPPH and SPCH cohorts. The ground truth for each image was delineated by experienced radiologists. **C** Schematic diagram of automated tumour segmentation (U-Net). The middle row of the network output represented the image segmented by the network, and the bottom row represented the ground truth. **D** Dice similarity coefficient (DICE) of trained automated segmentation model in the training and external test cohorts (large size: ≥ 2cm, small size: < 2cm). The subgroup analysis of DICE was also performed in Phase 0, Phase 1, Phase 2 (**E**), and different tumour sizes (**F**). **G** Images were segmented by the automated segmentation model for feature assessment. Three signatures (P0-Signature, P1-Signature and P2-Signature) were generated and further applied to build the SUAS combined with clinical factors. The performance of SUAS in distinguishing the pathological response (pCR vs. NpCR) was validated in two external test cohorts. *Abbreviations:* NAC: neoadjuvant chemotherapy; YNCH: Yunnan Cancer Hospital; GPPH: Guangdong Provincial People’s Hospital; SPCH: Shanxi Province Cancer Hospital; DICE: Dice similarity coefficient; SUAS: serial ultrasonography assessment system; pCR: pathological complete response; NpCR: non-pathological complete response

Eligible female patients diagnosed with breast cancer who completed NAC, followed by surgery, were retrospectively recruited from May 2015 to June 2020 according to the inclusion and exclusion criteria (Additional file 1: SI and Fig. S1). Serial ultrasonographic images of the target lesions were acquired at three time points: (1) pretreatment ultrasonography, within one week before NAC (Phase 0); (2) early-stage ultrasonography, during the first–second cycle of NAC (Phase 1); and (3) posttreatment ultrasonography, after NAC and within 1 month before surgery (Phase 2) (Fig. 1A). The cross-sectional image slice with the largest dimension of the tumour was selected for subsequent analysis. All images were reviewed by a radiologist with 10 years of experience in breast imaging (Y.L.). Details of the ultrasound scanners and probes used in the three centres are summarized in Additional file 1: Table S1.

Due to the retrospective nature of this study, the patients were not randomized into different cohorts. Patients enrolled from YNCH were divided into the training and internal validation cohorts between May 2015 and June 2018 and between July 2018 and June 2020 because this institute had the largest number of cases, while others recruited from GPPH and SPCH were used as two independent external test cohorts (Additional file 1: Fig. S1).

### Immunohistochemical evaluation

The oestrogen receptor (ER)/progesterone receptor (PR) status was considered positive if ≥ 1% of tumour cells were positive in immunohistochemical (IHC) staining [23]. For Ki-67 status, the cut-off values were < 20% and ≥ 20%. The human epidermal growth factor receptor 2 (HER2) status was considered positive if IHC was scored as 3+ , and negative if it was 0 or 1+ . In situ hybridization (ISH) was employed for cells with IHC scores of 2+ , and the HER2 status was considered positive with amplified result and negative with nonamplified results [24, 25].

### Assessment of pathological response to NAC

Six or more cycles of taxane-, anthracycline-, or anthracycline and taxane-based NAC protocols were administered to all patients (Table 1) according to the National Comprehensive Cancer Network (NCCN) and China Anti-Cancer Association breast cancer guidelines [10, 26]. For HER2(+) patients, an additional prescription of trastuzumab (8 mg/kg loading dose, 6 mg/kg maintenance dose) was given. Some of the HR(+)/HER2(−) patients received exclusive neoadjuvant endocrine therapy at the same time according to the recommendation.

**Table 1:** Patients clinicopathological characteristics in the four cohorts

Characteristics	Training cohort (*n* = 242)		*p*-value	Internal validation cohort (n = 197)		*p*-value	External test cohort1 (*n* = 212)		*p*-value	External test cohort2 (*n* = 150)		*p*-value
	pCR (*n* = 71)	NpCR (*n* = 171)		pCR (*n* = 57)	NpCR (*n* = 140)		pCR (*n* = 77)	NpCR (*n* = 135)		pCR (*n* = 38)	NpCR (*n* = 112)	
Age			0.4			0.4			0.4			0.7
< 40	19 (27%)	35 (20%)		9 (16%)	25 (18%)		10 (13%)	26 (19%)		5 (13%)	18 (16%)	
40–50	30 (42%)	69 (40%)		27 (47%)	65 (46%)		34 (44%)	50 (37%)		10 (26%)	35 (31%)	
> 50	22 (31%)	67 (39%)		21 (37%)	50 (36%)		33 (43%)	59 (44%)		23 (61%)	59 (53%)	
Menstruation			0.3			0.6			0.6			0.069
No	55 (77%)	122 (71%)		40 (70%)	101 (72%)		41 (53%)	67 (50%)		17 (45%)	69 (62%)	
Yes	16 (23%)	49 (29%)		17 (30%)	39 (28%)		36 (47%)	68 (50%)		21 (55%)	43 (38%)	
Ki-67			0.4			0.2			0.2			0.058
Negative	16 (23%)	48 (28%)		10 (18%)	43 (31%)		16 (21%)	38 (28%)		2 (5.3%)	20 (18%)	
Positive	55 (77%)	123 (72%)		47 (82%)	97 (69%)		61 (79%)	97 (72%)		36 (95%)	92 (82%)	
ER			0.2			< 0.001			< 0.001			0.003
Negative	26 (37%)	48 (28%)		21 (37%)	35 (25%)		51 (66%)	38 (28%)		21 (55%)	32 (29%)	
Positive	45 (63%)	123 (72%)		36 (63%)	105 (75%)		26 (34%)	97 (72%)		17 (45%)	80 (71%)	
PR			0.4			< 0.001			< 0.001			< 0.001
Negative	22 (31%)	44 (26%)		17 (30%)	33 (24%)		58 (75%)	57 (42%)		31 (82%)	55 (49%)	
Positive	49 (69%)	127 (74%)		40 (70%)	107 (76%)		19 (25%)	78 (58%)		7 (18%)	57 (51%)	
Her2			0.043			< 0.001			< 0.001			0.5
Negative	34 (48%)	106 (62%)		31 (54%)	100 (71%)		23 (30%)	80 (59%)		21 (55%)	69 (62%)	
Positive	37 (52%)	65 (38%)		26 (46%)	40 (29%)		54 (70%)	55 (41%)		17 (45%)	43 (38%)	
NAC protocols			0.3			0.068			0.068			0.15
Anthracycline-based	47 (66%)	110 (64%)		43 (75%)	92 (66%)		29 (38%)	55 (41%)		4 (11%)	28 (25%)	
Taxane-based	10 (14%)	36 (21%)		9 (16%)	27 (19%)		42 (55%)	56 (41%)		7 (18%)	14 (12%)	
Anthracycline and Taxane-based	14 (20%)	25 (15%)		5 (8.8%)	21 (15%)		6 (7.8%)	24 (18%)		27 (71%)	70 (62%)	
Subtype			0.10			< 0.001			< 0.001			0.004
HER2(+)	37 (52%)	64 (37%)		26 (46%)	40 (29%)		54 (70%)	55 (41%)		17 (45%)	45 (40%)	
Triple-negative	7 (9.9%)	19 (11%)		6 (11%)	17 (12%)		16 (21%)	17 (13%)		12 (32%)	13 (12%)	
Her2(−)&HR( +)	27 (38%)	88 (51%)		25 (44%)	83 (59%)		7 (9.1%)	63 (47%)		9 (24%)	54 (48%)	
Diameter (mean ± sd)	3.438 ± 1.345	3.604 ± 1.285	0.378	3.182 ± 1.364	3.795 ± 1.272	0.064	2.121 ± 1.387	2.974 ± 2.232	< 0.0001	1.926 ± 0.955	2.291 ± 0.963	0.056
P0-signature	− 0.16 (1.75)	− 1.37 (1.44)	< 0.001	− 0.14 (2.60)	− 1.69 (1.89)	< 0.001	0.59 (5.02)	− 2.73 (3.20)	< 0.001	− 0.4 (3.4)	− 4.0 (10.3)	< 0.001
P1-signature	− 0.09 (1.49)	− 2.95 (5.43)	< 0.001	0.12 (2.77)	− 2.42 (4.78)	< 0.001	0.7 (3.0)	− 3.0 (6.2)	< 0.001	0.4 (1.3)	− 3.1 (6.5)	< 0.001
P2-signature	0.13 (1.33)	− 2.77 (3.17)	< 0.001	1.0 (1.6)	− 2.3 (3.9)	< 0.001	1.8 (2.0)	− 2.8 (4.3)	< 0.001	0.6 (1.6)	− 2.4 (3.5)	< 0.001
Radiologist interpretation			0.030			< 0.001			< 0.001			< 0.001
NpCR	59 (83%)	158 (92%)		48 (84%)	128 (91%)		47 (61%)	118 (87%)		21 (55%)	112 (100%)	
pCR	12 (17%)	13 (7.6%)		9 (16%)	12 (8.6%)		30 (39%)	17 (13%)		17 (45%)	0 (0%)	

Data were mean (SD) or n (%). Chi-square (χ2) or Fisher’s exact tests were used to test whether the variable composition varied significantly between pCR and NpCR patients. A p value < 0.05 indicated that the variable distribution varied significantly between pCR and NpCR patients

*ER* estrogen receptor, *PR* progesterone receptor, *HER*2 human epidermal growth factor receptor, *NAC* neoadjuvant chemotherapy, *P0* Phase 0 (pretreatment), *P1* Phase 1 (early-stage treatment, namely, during the first–second cycle of the neoadjuvant chemotherapy), *P2* Phase 2 (posttreatment), *pCR* pathological complete response, *NpCR* non-pathological complete response

The postoperative assessment of pathological response was performed based on the American Joint Committee on Cancer staging system [27, 28]. pCR status was defined as no residual invasive disease in the breast and lymph nodes(with or without ductal carcinoma in situ)(ypT0/isypN0). All the specimens were evaluated by pathologists (with at least 9 years of experience).

To compare the predictive performance between the constructed models (see below) and radiologist interpretation, a board-certified radiologist with 10 years of experience (Y.L), who was blinded to the clinical records, independently reviewed the posttreatment ultrasound images, and patients without visible target lesions in the ultrasound image were classified as pCR [29].

### Tumour segmentation

Manual tumour segmentation is a time-consuming task, especially for ultrasound images that are affected by acoustic interference, signal attenuation, and artefacts, which may potentially increase the difficulty of manual segmentation. We proposed a deep learning segmentation model based on the 2D U-Net to achieve automated tumour segmentation (Fig. 1B, C). The regions of interest (ROIs) were manually delineated using itk-SNAP (www.itksnap.org) to obtain the ground truth by a trained radiologist (M.L, with 11 years of experience), then, an expert radiologist (Y.W, with 16 years of experience) confirmed the ROIs. In cases of disagreement, the ROI was adjudicated by a senior radiologist (Y.X.W, with 20 years of experience). The tumour ROI included the surrounding chords and burrs. If the tumour lesion was not visible after the NAC, the tumour bed fibrosis, the biopsy marker, and/or surrounding anatomic landmarks before NAC were used as the reference for ROI placement.

The segmentation network was based on the U-Net architecture proposed by Ronneberger [30]. The architecture consisted of two parts: (1) the encoding network, consisting of cascaded convolutional layers, maximum pooling layers, and full convolutions with skip connections, the purpose of which was to reduce the resolution of the input images and extract progressively abstract features; and (2) the decoding network, composed of a convolutional layer and an upsampling layer, the purpose of which was to offer an expanding path for resuming the spatial resolution of the extracted feature map to the original level of the input image (Additional file 1: Fig. S2). The details are provided in Additional file 1: SI II–III and Fig. S2. We used the Dice similarity coefficient (DICE) to evaluate the accuracy of automated segmentation.

### Feature extraction and signature construction

Since ultrasound images were collected from different image acquisition machines at multiple centres, the intensity distribution of the images was quite different. We first used the Z-Score method to standardize ultrasound images before extracting image features. Then, we further used the cosine similarity and Bland‒Altman plots to compare the similarity and consistency between the image features that were extracted from automated and manual segmentation by the Pyradiomics toolkit. Next, the ComBat model was used to reduce the batch effect caused by the images acquired by different machines. Finally, singular value decomposition and reconstruction (SVD-R), XGBoost, and support vector machine–recursive feature elimination (SVM-RFE) algorithms were used to execute feature selection. The details are provided in Additional file 1: SI IV–VI (Fig. 1G, Additional file 1: Fig. S3).

After feature selection, the optimal feature sets with correlations with pCR were selected for phase 0 (P0), phase 1 (P1), and phase 2 (P2). They were further used to build distinct single-time point prediction signatures (P0-Signature, P1-Signature, P2-Signature) by multivariable logistic regression.

### Model development and validation

Each single time point prediction signature generated a prediction score for each patient, which reflected the new characteristics of the tumour at different time points. Then, three prediction scores and clinicopathological factors were applied to generate four models to form the SUAS (Fig. 1G) to predict the pCR of patients receiving NAC: (1) the clinicopathological prediction model, which was built based on clinicopathological factors; (2) Model 1, which was built based on the P0-Signature and potentially significant clinicopathological factors; (3) Model 2, an early-stage treatment model, which was built based on Model 1 plus the P1-Signature; and (4) Model 3, which was built based on Model 2 plus the P2-Signature.

The application of SAUS was also investigated in the three molecular subtypes of breast cancer, namely, the HER2 (+),HER2(−)/HR(+), and triple-negative subgroups. To further validated the performance of models, we merged three datasets into one superset and then randomly split into training-validation-test cohort with a ratio of 6:2:2 (training cohort: *n* = 481, validation cohort: *n* = 160 and test cohort: *n* = 160). Then, we evaluated the predictive performance of the P0-Signature, P1-Signature, P2-Signature and SUAS model in each cohort.

### Statistical analysis

Continuous variables were expressed as the mean ± standard deviation (SD) or medians with interquartile range (IQR), as appropriate. Continuous and categorical variables were compared between groups utilizing Student's *t* test or the *χ*2 test. All statistical analyses were executed in R (version 3.5.0). A *p* value < 0.05 was considered statistically significant, and all tests were two-sided.

The cosine similarity, Bland‒Altman analysis and intraclass correlation coefficient (ICC) were used to analyse the similarity, consistency and agreement of the image features from automated segmentation and manual segmentation. The area under the receiver operating characteristic curve (AUC) and other performance evaluation metrics (Brier score, accuracy, sensitivity, specificity, positive predictive value [PPV], and negative predictive value [NPV]) were used to compare the performance between models (clinicopathological model and Models 1–3) and human interpretation. The 95% confidence intervals (95% CIs) were calculated using the bootstrapping strategy (*n* = 2000). DeLong's test, decision curves, the net reclassification improvement (NRI) test, and the integrated discrimination improvement (IDI) test were applied to assess the predictive performances of the models.

## Results

### Clinicopathological characteristics

In total, there were 1395 consecutive female patients potentially eligible for enrolment in the present study (YNCH, 718 patients; GPPH, 329 patients; SPCH, 348 patients), and 594 patients (YNCH, 279 patients; GPPH, 117 patients; SPCH, 198) were excluded. Therefore, 801 female patients (mean age 48 ± 9 years, range 25–75 years) were included in the final study cohorts, with 242 (YNCH), 197 (YNCH), 212 (GPPH), and 150 (SPCH) patients in the primary, internal validation and external test cohorts (Additional file 1: Fig. S1). Table 1 summarizes the clinicopathological characteristics of all patients.

There was no significant difference in the pCR rates among the primary, internal validation, and external test cohorts (29.3% vs. 28.9% vs. 36.2% [GPPH] vs. 25.3% [SPCH], *p* = 0.059). No significant difference in age, menstruation status, Ki-67 status, or NAC protocols (all *p* > 0.05) was observed between the pCR and NpCR groups in any of the four cohorts. In addition, ER and PR status were found to be significantly correlated with pCR in all cohorts except the training cohort. Her2 status showed no significant difference only in external test cohort 2.

### Deep learning enables comparability between automated and manual tumour segmentation.

The deep learning segmentation network we trained achieved satisfactory segmentation accuracy (DICE > 0.750) in two external test cohorts (Fig. 1D). During NAC, the residual tumours may become scattered foci distributed within the tumour bed [31], posing a great challenge for precise manual segmentation. However, our model demonstrated good segmentation accuracy for the images throughout the three phases (DICE > 0.780) (Fig. 1E). Meanwhile, our results showed that the automated model could perform effective segmentation not only for large-sized, but also for small-sized lesions (less than 2 cm) (Fig. 1F).

In addition, the similarity and consistency of the image features segmented by the two methods were favourable. A total of 3535 quantitative image features were extracted from automated and manual segmentation. According to the cosine similarity (mean > 0.900, range: 0.700–1.00) and Bland‒Altman test (mean difference ≤ 5.525e−11), the automated segmentation model demonstrated very close results to the manual segmentation performed by experienced radiologists in each cohort (Additional file 1: SI VI, Table S2). Therefore, we used the automatically segmented image features to complete the subsequent analysis.

### Feature assessment and SUAS construction and validation

Principal component analysis (PCA) and linear models show that Combat model indeed corrects the batch effect of machines (Additional file 1: Fig. S4). With the feature selection strategies, 12, 11, and 9 features were finally selected from phases 0–2 to build the P0-Signature, P1-Signature, and P2-Signature, respectively (Additional file 1: Table S3). The three signatures were significantly different between the pCR and NpCR groups and were important predictors for predicting pCR at multiple time points (Table 1, all *p* values < 0.001); however, unexpectedly, none of the clinicopathological factors, namely, ER, PR, HER2, Ki-67 status, were found to be significant in the multivariate regression analysis (Additional file 1: Table S4, all *p* values > 0.100). However, since previous studies have shown that these variables are important biomarkers for pCR prediction [7, 32], they were also built into the clinicopathological prediction model using the forced entry method of regression analysis. Furthermore, we observed that the relative contribution of each clinicopathological factor (0.77–13.63%) in Models 2–3 to predict pCR was much smaller than that of the radiomics signatures (25.21–46.85%) (Additional file 1: Table S4 and Fig. S5). Therefore, the clinicopathological factors were discarded in Models 1–3.

**Fig. 2:**
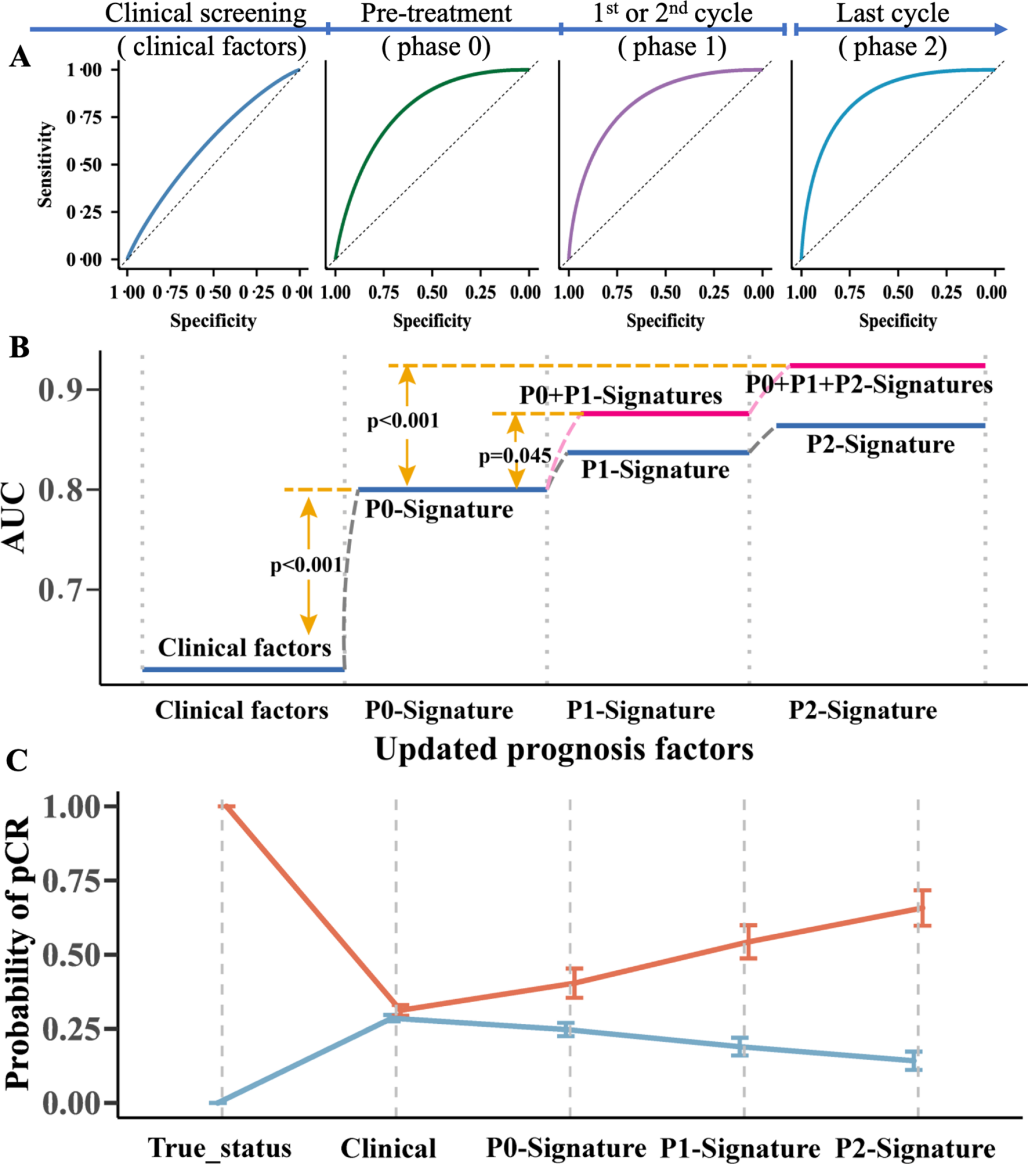
Evaluation of the performance of SUAS. **A** The ROC curves for each constituent part (clinical factors, Phase 0, Phase 1, and Phase 2) of SUAS which indicated the prediction performance for pathological response (pCR vs. NpCR) in the training cohort. **B** The AUC values of SUAS for pathological response prediction at each phase were illustrated with blue lines, while the incremental AUCs of SUAS when the signature of a new phase was accumulated onto signatures of previous phases were illustrated with pink lines. A significant increase in pCR prediction performance was observed in the latter (*p* values < 0.05). **C** The probability and 95% CI in predicting the pathological response for each constituent part of SUAS (the orange line for the pCR group and the steel-blue line for the NpCR group). *Abbreviations*: SUAS: serial ultrasonography assessment system; ROC curve: receiver operator characteristic curve; pCR: pathological complete response; NpCR: non-pathological complete response; AUC: area under the curve.

**Fig. 3:**
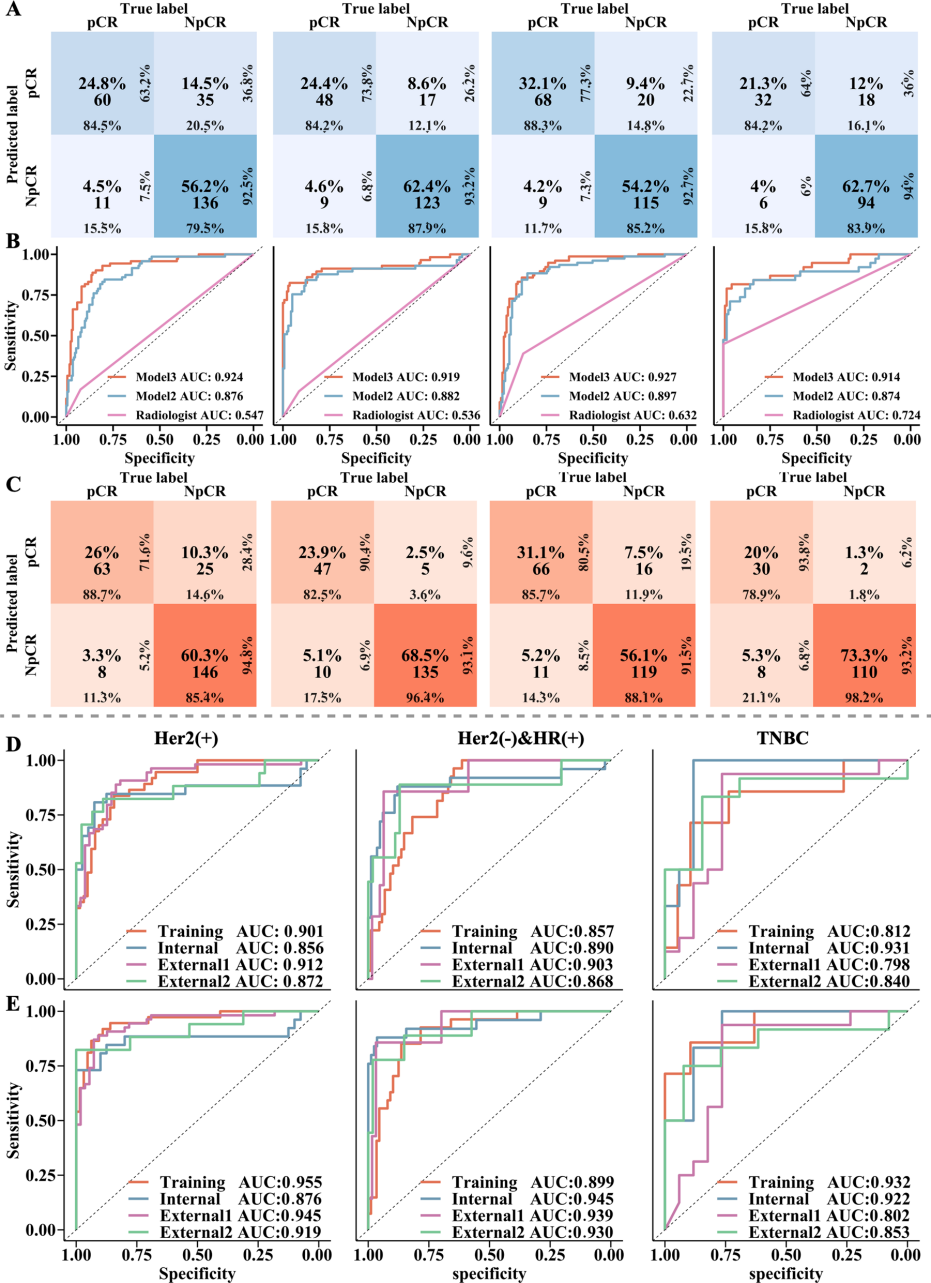
The performance evaluation of early-stage treatment model (Model 2) and posttreatment model (Model 3) in SUAS in all cohorts and subtypes of breast cancer. **A**, **C** Confusion matrices for Model 2 and Model 3 in the training and test cohorts. The number of cases correctly predicted, the percentage, sensitivity, specificity, positive predictive values and negative predictive values of each category was marked diagonally. **B** Area under the receiver operating characteristic curves for Model 2, Model 3 and radiologist in the training and test cohorts. **D**, **E** The predictive performance of Model 2 (**D**) and Model 3 (E) in the three subgroups, namely the Her2(+), the Her2(−)&HR(+), and the TNBC subgroup. *Abbreviations*: SUAS: serial ultrasonography assessment system; HR: hormone receptor; HER2: human epidermal growth factor receptor 2; TNBC: triple negative breast cancer.

The performance of the single time point signature in predicting pCR was significantly superior to that of the clinical model (*P*_all_ < 0.001) and radiologist evaluation (*P*_all_ < 0.001) (Additional file 1: Table S5). Similar results were found in Models 2–3 for clinical usefulness (Additional file 1: Fig. S6). Furthermore, adding the single time point signature to another signature to form a multitime point model significantly improved the prediction for pCR (Fig. 2A, B, Table 2, Additional file 1: Table S6). Additionally, in the evaluation of the relative variable contribution to SUAS, the highest percent contribution was the posttreatment signature (46.16%), followed by the pretreatment signature (25.21%), early-stage treatment signature (16.22%), and clinical factors (12.4%) (Additional file 1: Fig. S5). In clinical practice, early assessment of treatment response can help assess the effectiveness of treatment options [6]. The early-stage treatment-based Model 2 (P0 + P1-Signatures) was superior to existing methods, such as clinical models or radiologists (Fig. 3A, B, Table 2). A similar result was detected in Model 3 (P0 + P1 + P2-Signatures), which aimed at preoperative evaluation after NAC (Fig. 3B, C, Table 2). Moreover, 468 of 558 (83.9%) patients with NpCR (136 of 171, 123 of 140, 115 of 135, and 94 of 112 patients in the training, internal validation, and two external test cohorts, respectively) were successfully identified by Model 2(Fig. 3A). Meanwhile, 206 of 243 (84.8%) patients with pCR (63 of 71, 47 of 57, 66 of 77, and 30 of 38 patients in the training, internal validation, and two external test cohorts, respectively) were successfully identified by Model 3 (Fig. 3C). Finally, the subgroup analysis of SUAS was implemented, and Models 2 and 3 achieved better predictive performance within the HER2(+) and HER2(−)/HR(+) subgroups (AUCs all > 0.860), than within the triple-negative subgroup (AUCs < 0.860) (Fig. 3D, E, Additional file 1: Table S6). The outperformance of SUAS was further validated by the NRI test (with all *p* < 0.001, Additional file 1: Table S7) and IDI test (with all *p* < 0.001, Additional file 1: Table S8). Besides, the superiority of SUAS in predicting pCR and NpCR status was also validated in randomly divided datasets (Additional file 1: Table S9).

**Table 2:** Multivariate analysis of clinicopathological model, Model 2 (the early-treatment model) and Model 3 (the posttreatment model)

	Clinicopathological model		*p*-value	Model 2		*p*-value	Model 3		*p*-value
	OR	95%CI		OR	95%CI		OR	95%CI	
Ki67									
Negative	Reference								
Positive	1.436	0.783–2.633	0.240	1.716	0.815–3.615	0.153	1.985	0.858–4.594	0.107
ER									
Negative	Reference								
Positive	0.742	0.333–1.657	0.465	0.559	0.212–1.470	0.236	0.700	0.242–2.024	0.509
PR									
Negative	Reference								
Positive	0.990	0.427–2.302	0.984	0.701	0.258–1.904	0.484	0.768	0.231–2.548	0.664
Her2									
Negative	Reference								
Positive	0.937	0.078–11.320	0.959	1.062	0.369–3.055	0.911	1.036	0.063–16.92	0.980
Subtype									
Triple-negative	Reference								
HER2(+)	2.047	0.161–25.97	0.578	1.375	0.083–22.90	0.823	1.271	0.075–21.44	0.867
HER2(−)&HR(+)	1.194	0.361–3.945	0.770	1.275	0.305–5.331	0.738	1.378	0.272–6.987	0.697
P0-Signature	–	–	–	2.357	1.531–3.629	< 0.001	1.881	1.222–2.894	0.004
P1-Signature	–	–	–	2.455	1.721–3.502	< 0.001	2.094	1.468–2.987	< 0.001
P2-Signature	–	–	–	–	–	–	2.097	1.543–2.849	< 0.001

Model 2 was built based on the P0-Signature plus the P1-Signature. And Model 3 was based on Model 2 plus the P2-Signature

*ER* estrogen receptor, *PR* progesterone receptor, *HER2* human epidermal growth factor receptor, *P0* Phase 0 (pretreatment), *P1* Phase 1 (early-stage treatment, namely, during the first–second cycle of the neoadjuvant chemotherapy), *P2* Phase 2 (posttreatment), *OR* odds ratio, *95%CI* 95% confidence interval

### Visualization and interpretability of SUAS

**Fig. 4:**
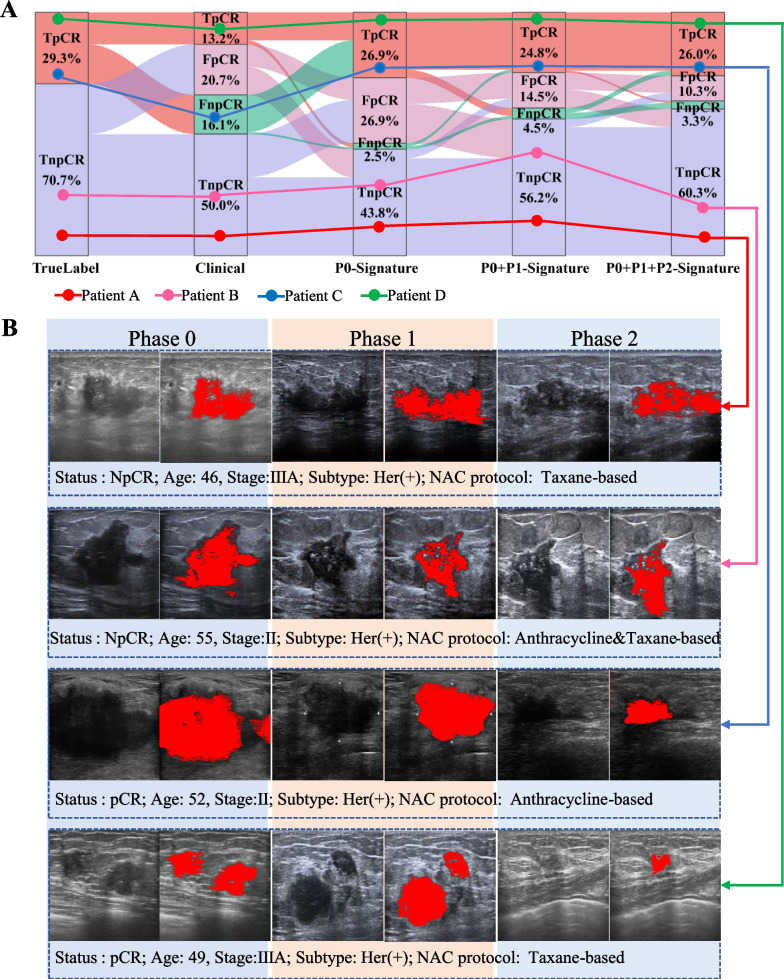
Visualization and interpretability of SUAS. **A** The Sankey diagram of changes among the TpCR, TnpCR, FpCR, and FnpCR in difference prognosis factors, with the increment of the predictors in SUAS, all categories (TpCR, FpCR, FnpCR, and TnpCR) presented with significant and constant fluidity. **B** Entropy, the feature extracted from the ultrasound images of four patients, was visualized in Phases 0–2. It could be observed that even for patients with pCR and NpCR who had the same tumour stage, the similar NAC protocol and age (Patient A vs Patient D, Patient B vs Patient C), the characteristics of the ultrasound images in Phases 0–2 were not apparently different by naked eyes. But after clustering the entropy in the tumour area, it could be clearly found that the clustering of NpCR patients was more scattered than that of pCR patients, with chords and burrs around. *Abbreviations:* SUAS: serial ultrasonography assessment system; TpCR: true positivity; TnpCR: true negativity; FpCR: false positivity; FnpCR: false negativity; pCR: pathological complete response

A Sankey Diagram (Fig. 4A) was employed to visualize the predictive accuracy of SUAS throughout NAC, which reflected the constituent proportions of pCR prediction performance (true positivity (TpCR), true negativity (TnpCR), false positivity (FpCR) and false negativity (FnpCR)) of SUAS for each prognostic factor. The diagram showed that with the increment of the predictors in SUAS, all categories (TpCR, FpCR, FnpCR, and TnpCR) presented significant and constant fluidity (e.g. from clinicopathological factors to the P0 signature). The largest shift was observed during the transition from the P0-Signature to the P0 + P1-Signatures, where the FpCR decreased by 12.4% and the TnpCR increased by 12.4%. Similar results were found during the transition from the P0 + P1-Signatures to the P0 + P1 + P2-Signatures. Briefly, with the accumulation of information on multiple prognostic factors, the proportion of false positivity prediction showed a downward trend, while the proportion of true negativity gradually increased.

We also quantified three entropy-related feature changes over time in the three institutions. The results revealed that patients with pCR showed reduced entropy, while those with NpCR showed the opposite (Additional file 1: Fig. S7).

Meanwhile, four patients (two with pCR and two without) were randomly chosen from the study population to explore the interpretability of SUAS by the clustering of entropy during NAC (Fig. 4B). In patients with NpCR, the entropy clustering within the tumour bed became increasingly scattered from Phase 0 to 2, with chords and burrs around. In patients achieving pCR, the entropy clustering within the tumour bed demonstrated increased compactness throughout the three phases, with a well-defined margin. This result suggested that the SUAS could interpret the potential biological changes in breast cancer throughout NAC.

## Discussion

This study showed that the autosegmentation-based SUAS, integrating serial multitime imaging biomarkers throughout NAC, could accurately predict pCR in the training, internal validation and two external test cohorts. Moreover, the performance of SUAS was largely unaffected by the molecular subtypes. To the best of our knowledge, this is the first large-sample, multicentre study that incorporated pre, early-stage, and posttreatment ultrasonographic imaging features. The outperformance of SUAS over the clinical model, human interpretation, and conventional single time-point prediction models indicated its potential in facilitating individualized clinical decision-making noninvasively before surgery in breast cancer patients.

The most essential finding of the present study was the importance of serial (rather than single time-point) assessment in predicting the pCR of breast cancer. Breast cancer is a group of highly heterogeneous neoplasms that evolve continuously over space and time [33]. In particular, the dynamic response to NAC may contain a large amount of information that is potentially associated with the pathological outcome. Therefore, how to track the full-scale changes during NAC and whether dynamic imaging profiling would contribute to the improvement of prediction performance have become the major concerns. In the present study, a trend towards an increase in performance with higher AUCs was noted, when new imaging signatures of different time-points throughout NAC were added to the model. This was especially evident in Model 3 which included the posttreatment signature. The relative variable contribution analysis also confirmed that the posttreatment signature contributed to 46.16% of the predictive power of SUAS, followed by the pretreatment signature (25.21%), early-stage treatment signature (16.22%), and clinical factors (12.4%). The two latest published studies also developed a deep learning radiomic model from serial ultrasonographic data to predict the treatment response to NAC in patients with breast cancer [12, 17]. However, Gu et al. [17] study mainly focused on early adjustment of the NAC treatment strategy; thus, ultrasonographic data were obtained before treatment and after the second and fourth courses. Jiang et al. [12] prediction model only focused on the preoperative prediction of pCR based on pre- and posttreatment ultrasonographic data to guide surgical options. Our work achieved accurate prediction of pCR not only in the early stage (AUC of 0.874 and 0.897 in two external validation cohorts) but also in posttreatment of NAC (AUC of 0.927 and 0.914 in two external validation cohorts) by integrating the pre, early-stage, and posttreatment information. In the present study, the early prediction model (Model 2) successfully identified 83.9% (468 of 558) of NpCR patients, who may benefit from adjusting the treatment regimen; moreover, the preoperative model (Model 3) successfully identified 84.8% (206 of 243) of the pCR patients who may benefit from breast-conserving surgery and the omission of axillary node dissection. In addition, both the internal and external validation cohorts were included in the present study, ensuring a more robust assessment of model performance. In addition, we did not employ the deep learning technique in model construction to avoid the so-called black-box phenomenon, so the results of our study were more explicable. We also searched MRI-based radiomics to predict treatment response in breast cancer. Most of them were based on single-time MRI features [7, 32, 34, 35] because it is difficult for patients to undergo repeat MRI examination at short time intervals. Given the accessibility and operational simplicity of ultrasonography, the feasibility of predicting pCR of breast cancer using serial ultrasonographic assessment warrants consideration in clinical practice.

Another finding of this research was that the developed deep learning segmentation model could enable automated tumour segmentation comparable to manual segmentation, with satisfactory consistency and agreement, which significantly decreased the annotation time for the application of SUAS in clinical settings. Manual segmentation of the tumours is a laborious task with potential intra- and interobserver variability, especially for a large amount of data obtained from multiple time-points throughout NAC. To facilitate the feasibility of SUAS, quantitative analysis should be as user-friendly as possible. Therefore, the automated segmentation method was developed with a deep learning network (U-net). Our findings suggest that SUAS has the potential to become an automatic tool for pCR assessment before surgery.

In SUAS, we identified 12, 11, and 9 different radiomics features from pre-, early-stage, and post-NAC ultrasound images to discriminate pCR and NpCR status. This suggested that the biomarkers of tumour could be changed during NAC. However, the biological interpretation of these features remains an area of active investigation [36]. A common entropy-related feature from serial images measured complexity of grey-level intensity (Entropy, Run Entropy) and heterogeneity of texture patterns (Zone Entropy), possibly reflecting the texture of cell proliferation and tissue hypoxia, which has been shown to be associated with response to neoadjuvant therapy [37]. Among the 32 features, 15 features (such as Large Dependence Low Grey-Level Emphasis) were related to the grey level of images, which evaluate overall and clustered low or high grey-level intensity values. Changes in greyscale may reflect fibrotic and aggressive growth of the tumour and are associated with poor treatment outcomes [38, 39]. Other features (such as clustershade, skewness) are indicators that measure grayscale intensity and texture uniformity, which reflecting the intratumoural heterogeneity and slight variation of tissue morphology within the tumour. In total, radiomic features evaluated in SUAS highlight tumour heterogeneity at a regional and local level, which, depending on types of feature matrix, could be linked with proliferation, angiogenesis, and necrosis.

Currently, clinician assessment of pCR by human interpretation of ultrasound images is limited due to insufficient accuracy, probably because the tumour response is more reflected by changes in pathological compositions and microenvironment, such as necrosis and fibrosis, rather than changes in size which can be readily perceived by the naked eye [12]. We compared the performance of the SUAS with that of clinicians for all datasets and found that the SUAS was far superior to the human experts. However, the predictive performance of clinicopathological factors was unfavourable in the external test cohorts, with a low contribution to the predictive power of the SUAS, probably because of the inconsistent distribution of molecular types across the four cohorts.

Since different molecular subtypes of breast cancer may result in variable responses to NAC, we also performed subgroup analysis to determine the performance of SUAS in the specific subtypes of breast cancer. First, our study suggested that the SUAS could accurately predict the pathological outcome in the HER2(−)/HR(+) subgroup, with the highest performance among the three subtypes (AUC = 0.954 in the external validation cohort), even without the posttreatment signature (AUC = 0.916 in the external validation cohort). Conventionally, the HER2(−)/HR(+) subtype is considered insensitive to NAC, with a pCR rate < 10% [3]. For patients with this subtype who have large tumours but still desire to conserve the breast, Model 2 of the SUAS can assist in the early determination of potential candidates that can truly benefit from NAC and avoid the unnecessary toxic effects of chemotherapy and cost of the treatment. Second, patientsinHER2(+) subgroup and the triple-negative group are well-known for their high probability of response to NAC [3]. However, the predictive performance of Model 2 was relatively unsatisfactory in the TNBC subgroup analysis of the GPPH external test cohort (AUC = 0.798), probably because the proportion of patients with TNBC in the training cohort was the lowest among the cohorts (only 7 patients, 9.9%). After integrating the posttreatment ultrasonographic signature into the prediction model, the AUC increased to 0.802, which implied the significance of serial ultrasonography assessment throughout NAC. Of note, the ultrasonography-based prediction model in our study outperformed the multiparametric MRI-based prediction model developed by Liu et al. [7] in most of the subgroup analyses, with AUCs ranging from 0.78 to 0.87 among the three external cohorts for the HER2(−)/HR(+) subgroup, 0.58–0.79 for the HER2(+) subgroup, and 0.79–0.84 for the triple-negative subgroup. This was unexpected because MRI has been considered the method of choice that provides the most correlated measurement of tumour size with pathological results [40]. A possible explanation is that the multiphase biological and pathophysiological changes during NAC captured by serial ultrasonography made a considerable contribution to the outcome prediction, which outweighed the information detected by single time-point pretreatment MRI.

There were several limitations of the present study. First, the distribution of most patient characteristics and NAC regimens were not balanced among the four cohorts, which may have a potential influence on the validation of the SUAS. However, our study showed that the AUCs of Models 1–3 were similar between the training cohort and the other three cohorts, which implied the general applicability of the SUAS in various clinical situations. Second, only the ultrasound images obtained during the first–second cycle of NAC were used for model construction, the purpose of which was to simplify the entire data procurement protocol and improve the feasibility of clinical evaluation. The relative variable contribution analysis showed that the pretreatment and posttreatment signatures made a greater contribution to the prediction performance of the SUAS. Third, the present SUAS was meant to be a preliminary tool for patient stratification, which should be applied with caution because of the retrospective nature of this study with inherent selection bias. Given the complexity of patients’ clinical situations, it should be noted that the surgical strategy must be based on the comprehensive assessment by the multidisciplinary team.

## Conclusions

In conclusion, the present proof-of-concept study developed a feasible model (the SUAS) based on automated segmentation of pre, early-stage, and post-NAC ultrasonographic imaging features for predicting patients with breast cancer who could benefit from optimal therapeutic management after NAC.

## Supplementary Information

**Additional file 1. SI**. Inclusion and exclusion criteria.** SII**. Annotation of ultrasound images.** SIII**. Details of theautomated segmentation model for breast ultrasound images.** SIV**. Data Standardization and Feature Extraction.** SV**. Feature Selection and Model Construction.** SVI**. Consistency evaluation of features by Bland-Altman.** Fig. S1**. Inclusion and exclusion criteria.** Fig. S2**. U-net architecture.** Fig. S3**. Flowchart of feature assessment and SUAS construction.** Fig. S4**. Principal component analysis plot and linear model for evaluating batch effect correction in three institutions.** Fig. S5**. Relative variable contribution in Model2 and Model3.** Fig. S6**. Clinical usefulness evaluation of Model2 and Model3.** Fig. S7**. The changing trend of entropy-related features in three institutions.** Table S1**. The ultrasonographic images acquisition parameters of the multiple centers.** Table S2**. Similarity and consistency evaluation between automated segmentation and manual segmentation.** Table S3**. Key features selected for construction of phasal signatures based on ultrasound images at each single time point.** Table S4**. Multivariate analysis of clinicopathological model, Model 2 (the early-treatment model) and Model 3 (the posttreatment model).** Table S5**. Prediction performance of the signatures.** Table S6**. AUCs of early-pretreatment model (Model 2) and post-treatment model (Model 3) in breast cancer subtypes.** Table S7**. NRI test for prediction improvements of SUAS compared to single-time signature in multiple cohorts.** Table S8**. IDI test for prediction improvements of SUAS compared to single-time signature in multiple cohorts.** Table S9**. Prediction performance of the signatures and SUAS in randomly split datasets.

## Data Availability

All data needed to evaluate the conclusions in the paper are present in the paper and/or the Additional file 1. Additional data related to this paper (including deidentified participant data with the data dictionary, original ultrasonographic images, study protocol and statistical analysis plan, etc.) will be made available to the scientific community on publication but should be reasonably requested from the corresponding authors. A signed data use agreement and institutional review board approval will be required before the release of research data.

## Abbreviations

SUAS: Serial ultrasonography assessment system
pCR: Pathological complete response
NAC: Neoadjuvant chemotherapy
AUC: Area under the receiver
ER: Estrogen receptor
PR: Progesterone receptor
IHC: Immunohistochemical
HER2: The human epidermal growth factor receptor 2
ROI: The region of interest
NRI: Net reclassification improvement
IDI: Integrated discrimination improvement
PPV: Positive predictive value
NPV: Negative predictive value
ICC: Intraclass correlation coefficient
