# Supplementary material for: An integrated deep learning model for the prediction of pathological complete response to neoadjuvant chemotherapy with serial ultrasonography in breast cancer patients: a multicentre, retrospective study
Source: Breast Cancer Res. 2022 Nov 21;24:81. doi: 10.1186/s13058-022-01580-6 (PMC9680135; doi:10.1186/s13058-022-01580-6)
Supplement: Supplementary file 1 — Additional file 1. SI. Inclusion and exclusion criteria. SII. Annotation of ultrasound images. SIII. Details of theautomated segmentation model for breast ultrasound images. SIV. Data Standardization and Feature Extraction. SV. Feature Selection and Model Construction. SVI. Consistency evaluation of features by Bland-Altman. Fig. S1. Inclusion and exclusion criteria. Fig. S2. U-net architecture. Fig. S3. Flowchart of feature assessment and SUAS construction. Fig. S4. Principal component analysis plot and linear model for evaluating batch effect correction in three institutions. Fig. S5. Relative variable contribution in Model2 and Model3. Fig. S6. Clinical usefulness evaluation of Model2 and Model3. Fig. S7. The changing trend of entropy-related features in three institutions. Table S1. The ultrasonographic images acquisition parameters of the multiple centers. Table S2. Similarity and consistency evaluation between automated segmentation and manual segmentation. Table S3. Key features selected for construction of phasal signatures based on ultrasound images at each single time point. Table S4. Multivariate analysis of clinicopathological model, Model 2 (the early-treatment model) and Model 3 (the posttreatment model). Table S5. Prediction performance of the signatures. Table S6. AUCs of early-pretreatment model (Model 2) and post-treatment model (Model 3) in breast cancer subtypes. Table S7. NRI test for prediction improvements of SUAS compared to single-time signature in multiple cohorts. Table S8. IDI test for prediction improvements of SUAS compared to single-time signature in multiple cohorts. Table S9. Prediction performance of the signatures and SUAS in randomly split datasets. [file 13058_2022_1580_MOESM1_ESM.docx]

**Supplementary Appendix**

**Supplementary Information**

**I. Inclusion and exclusion criteria**

**II. Annotation of ultrasound images**

**III. Details of the automated segmentation model for breast ultrasound images**

**IV. Data Standardization and Feature Extraction**

**V. Feature Selection and Model Construction**

**VI. Consistency evaluation of features by Bland-Altman**

**Supplementary Figures**

**Figure S1. Inclusion and exclusion criteria**

**Figure S2. U-net architecture**

**Figure S3. Flowchart of feature assessment and SUAS construction**

**Figure S4. Principal component analysis plot and linear model for evaluating batch effect correction in three institutions.**

**Figure S5. Relative variable contribution in Model2 and Model3**

**Figure S6. Clinical usefulness evaluation of Model2 and Model3**

**Figure S7. The changing trend of entropy-related features in three institutions.**

**Supplementary Tables**

**Table S1. The ultrasonographic images acquisition parameters of the multiple centers**

**Table S2. Similarity and consistency evaluation between automated segmentation and manual segmentation**

**Table S3. Key features selected for construction of phasal signatures based on ultrasound images at each single time point**

**Table S4. Multivariate analysis of clinicopathological model, Model 2 (the early-treatment model) and Model 3 (the post-treatment model)**

**Table S5. Prediction performance of the signatures**

**Table S6. AUCs of early-pretreatment model (Model 2) and post-treatment model (Model 3) in breast cancer subtypes**

**Table S7. NRI test for prediction improvements of SUAS compared to single-time signature in multiple cohorts**

**Table S8. IDI test for prediction improvements of SUAS compared to single-time signature in multiple cohorts**

**Table S9. Prediction performance of the signatures and SUAS in randomly split datasets.**

**Reference**

**Supplementary Information**

**I. Inclusion and exclusion criteria**

The inclusion criteria included: (1) patients with biopsy-proven unilateral primary invasive breast cancer; (2) patients who received complete NAC without history of cancer treatment; (3) the pathological outcomes after NAC were confirmed by surgery; (4) ultrasound images obtained before, during the first or second cycle, and after NAC were available. The exclusion criteria were: (1) patients who failed to receive standard NAC (mainly referring to absence of trastuzumab administration in HER2-positve patients); (2) ultrasound images of poor quality; (3) patients without complete clinical and pathological information.

**II. Annotation of ultrasound images**

The region of interest regions (ROIs) were manually delineated using itk-SNAP ([www.itksnap.org](http://www.itksnap.org)) to obtain the ground truth by a trained radiologists (M.L, with 11 years of experience), then, an expert radiologists (Y.W, with 16 years of experience) confirmed the ROIs. In case of disagreement, the ROI was adjudicated by a senior radiologist (Y.X.W, with 20 years of experience). The tumor of ROI included the surrounding chords and burrs. If the tumor lesion was no visible after the NAC, the tumor bed fibrosis, the biopsy marker, and/or surrounding anatomic landmarks before NAC were used as the reference for ROI placement.

**III. Details of the automated segmentation model for breast ultrasound images**

**i) Ground Truth**

All images were manually outlined by a sonographer and reviewed by an experienced doctor (shown in the Supplementary Methods II).

**ii) Network Architecture**

The segmentation network was based on the U-Net architecture proposed by Ronneberger.[^1^](#_ENREF_1) The architecture consisted of two parts: 1) the Encoding network, consisting of cascaded convolutional layers, maximum pooling layers, and full convolutions with skip connections, the purpose of which was to reduce the resolution of the input images and extract progressively abstract features; 2) the Decoding network, composed of a convolutional layer and an up-sampling layer, the purpose of which was to offer an expanding path for resuming the spatial resolution of the extracted feature map to the original level of the input image (shown in Supplementary Figure S2).

**iii) Implementation Details**

We implemented the automated segmentation model with Python v3.6.13 (<https://www.python.org/>) using Tensorflow (version2.4.0, Google, Menlo Park, CA, USA) and Keras (version2.4.3, Francois Chollet, Menlo Park, CA, USA) libraries in a workstation with an RTX2080Ti GPU. The loss function was defined by Dice coefficient:

$$loss\left( I_{G},I_{P} \right)=1-\frac{2I_{G}*I_{P}+1}{I_{G}+I_{P}+1}$$

Where, $I_{G},I_{P}$ denoted images of ground truth and predicted masks, respectively.

We utilized dropout regularization of 0.6 to avoid overfitting. Xavier normal initialization was used to randomly initialize the network weights. Adaptive moment estimation optimizer (Adam) with a learning rate (LR) of 10e-4, and a batch size set to 16 were applied to train all parameters in the training cohort. The training was stopped when the loss on the validation cohort did not decrease for 50 training epochs.

**iv) Evaluation metrics**

We utilized dice similarity coefficient (DICE), a commonly used index in segmentation models, to evaluate the segmentation performance of the model:

$$DICE=\frac{2*abs(P_{g}\cap P_{P})}{abs\left( P_{g} \right)+abs(P_{P})}$$

Where,$P_{g}$ was the set of pixels that included tumor region in the ground truth, and $P_{P}$ was the set of pixels that included the tumor region segmented by the model. The closer the DICE value was to 1, the better the segmentation result.

**v) Segmentation Results.**

The U-Net model performed well with the average dice similarity coefficients of 0.806 and 0.785 for external test 1 and external test 2 cohorts, respectively. This model performed well for large tumors (max diameter >1 cm), with the DICEs of 0.818 and 0.795 for external test 1 and external test 2 cohorts, respectively.

**IV. Data Standardization and Feature Extraction**

In order to compare the differences between the automated and manual segmentation, we extracted the features of the regions of interest based on the results of the two methods at the same time (denoted as: Auto-F, features extracted from auto-segmentation; Manu-F for features extracted from manu-segmentation). Since ultrasound images were collected from different image acquisition machines in multiple centers, and the intensity distribution of the images was quite different. To reduce the impact of image differences on the results, we adopted the following strategies:

**Step 1**: At the image level, we used the general image standardization algorithm, z-score, to standardize the images from different centers and machines. The normalized value of the image intensity was computed as follows:

$$z=\frac{X-\mu}{\sigma}$$

After z-score normalization of the pixel intensities of each image, 3535 quantitative ultrasound features were extracted using the Pyradiomics toolkit (<https://github.com/AIM-Harvard/pyradiomics>) based on Python (Version 3.6.13), including: 1) 18 first-order statistical features used to reflect the tumor intensity, such as entropy, energy, etc.; 2) nine morphological features describing the tumor morphology, such as diameter, area, etc.; 3) 68 texture features used to describe the tumor heterogeneity; and 4) 3440 wavelet features of tumor heterogeneity were described in terms of frequency and direction by performing wavelet transformation on the original image. In total, 10605 features were extracted from Phase 0, Phase1 and Phase 2 image for each patient. The mathematical definition and description of imaging features can be found in van Griethuysen et al.’s study[^2^](#_ENREF_2) or viewed on the PyRadiomics website. (https://pyradiomics.readthedocs.io/en/latest/).

**Step 2**: At the feature level, we used the ComBat model for the harmonization of multi-centre and multi-machine features from step one. The ComBat model was originally developed for gene expression microarray data by Johnson et al.[^3^](#_ENREF_3) Here, we referred to the study of Fortin et al.,[^4^](#_ENREF_4)^,^ [^5^](#_ENREF_5) and reformulated the ComBat model in feature processing. We supposed that the features generated from $c$ centers, each center containing $m_{i}$ machine, where $i$=1, 2… For feature $f$ =1, 2, 3, we used $y_{ijf}$ to represent the feature for machine $i$ in center $j$. According to the study of Johnson et al.,[^3^](#_ENREF_3) Combat model was defined as:

$$y_{ijf}=\alpha_{f}+X\beta_{f}+\gamma_{if}+\delta_{if}\varepsilon_{ijf}$$

Where $\alpha_{f}$ represented the generated feature, $X$ was a matrix designed according to the samples, and $\beta_{f}$ was a regression coefficient related to $X$. $\gamma_{if}$ and $\delta_{if}$ represented the additive and multiplicative center effects. $\varepsilon_{ijf}$ was a noise term, which was supposed to conform to a normal distribution with a mean of zero and a variance of $\sigma_{f}^{2}$.

Further, assuming that the effect parameters had parameters with prior distributions, the empirical bayes was used to evaluate the variance of the parameter estimates $\hat{\gamma}_{if}$ and $\hat{\delta}_{if}$. According to the description of a previous article:[^3^](#_ENREF_3)

$$\gamma_{if}\sim N(\gamma_{i}, {(\tau_{i})}^{2}$$

$${(\delta_{if})}^{2}\sim InverseGamma(\lambda_{i}, \theta_{i})$$

Finally, the ComBat model could be defined as

$$y_{ijf}^{ComBat}=\frac{y_{ijf}-\hat{\alpha}_{f}-X\hat{\beta}_{f}-\gamma_{if}^{*}}{\delta_{if}^{*}}+\hat{\alpha}_{f}+X\hat{\beta}_{f}$$

**V. Feature Selection and Model Construction**

Before performing feature selection, we evaluated the similarity and consistency between Auto-F and Manu-F. First, the similarity between Auto-F and Manu-F of each patient was evaluated by cosine similarity; then, Bland-Altman was used to evaluate the consistency of each feature of Auto-F and Manu-F. Finally, intraclass correlation coefficient (ICC) with threshold of p-value at 0.75 was applied to select the features with high agreement from Auto-F were used to downstream analysis. To select important features related to breast cancer neoadjuvant treatment response from the features, the following strategies were adopted:

**(1) Singular value decomposition and reconstruction**. First, singular value decomposition (SVD) took a feature matrix (defined as $F$, where $F$ was a $n\times p$ matrix) in which the $n$ rows represented the patients, and the $p$ columns represented the image features. According to the SVD theorem:

$$F_{n\times p}=U_{n\times n}\Sigma_{n\times p}V_{p\times p}^{T}$$

$U$ was a complex unitary matrix, $\Sigma$was a rectangular diagonal matrix, and $V$ was a complex unitary matrix.

Then, the original feature matrix (defined as $F^{r}$ ) was reconstructed by decomposed matrix $U$, $\Sigma$ and $V$

$$U_{n\times n}\Sigma_{n\times p}V_{p\times p}^{T}=F_{n\times p}^{r}$$

Due to the loss of information in the process of matrix decomposition and reconstruction, only part of the information could be completely reconstructed. In other words, $F_{n\times p}^{r}$was not equal to $F_{n\times p}$. In this process, the information that could be reconstructed was regarded as useful information. And the intraclass correlation coefficient (ICC) was used with threshold of p-value set to 0.75 to select the main features.

**(2) XGBoost**. XGBoost is a gradient boosting decision tree which performed the second-order Taylor expansion, which could combine multiple weak learners to generate a strong learner, and control the complexity of the tree and increase the regularization term to suppress model overfitting and quickly improve the model performance.[^6^](#_ENREF_6) In our research, gain was used to determine the optimal split node.

$$gain=\frac{{(\sum_{i\in I_{l}} g_{i})}^{2}}{2(\sum_{i\in I_{l}} h_{i}+\lambda)}+\frac{{(\sum_{i\in I_{r}} g_{i})}^{2}}{2(\sum_{i\in I_{r}} h_{i}+\lambda)}-\frac{\left( \sum_{i\in I} g_{i} \right)^{2}}{2(\sum_{i\in I} h_{i}+\lambda)}-\gamma$$

Where $g_{i}$, $h_{i}$ represented the 1st- and 2nd-order gradients. $I_{l}$, $I_{r}$ denoted left and right nodes after split. $\lambda$ and $\gamma$ were the penalty factors. $I=I_{l}\cup I_{r}$.

The gain score generated by each split of a tree could be calculated by the above formula, and the final feature importance was determined by the average gain. The higher the feature importance score was, the more effective or relevant the corresponding feature was. The R version of the XGBoost package can be obtained at <https://cran.r-project.org/web/packages/xgboost/index.html>.

**(3) Support Vector Machine–Recursive Feature Elimination (SVM-RFE)**. SVM-RFE is an embedded feature selection method that based on SVM algorithm.[^7^](#_ENREF_7) During performance, it would remove features recursively according to the smallest ranking criterion until all features are removed.

By this way, 12 Phase 0 features, 11 Phase1 features and nine Phase2 features, were selected to build three distinct single-time prediction signatures by logistic regression in the training cohort, namely, the Phase 0 signature (P0-signature), the Phase1 signature (P1-signature), and the Phase2 signature (P2-signature), respectively. Each single-time prediction signature would generate a prediction score for each patient, which reflected the new characteristics of the tumor at different time points. Then, three prediction scores and clinicopathological factors were used to build a multi-time prediction model, namely, the serial ultrasonography assessment system (SUAS). Furthermore, since evaluating the treatment response in the early stage of neoadjuvant treatment is crucial in adjusting treatment strategies, we used the prediction scores of two single time-point, Phase 0 and Phase 1, to construct an early prediction model (namely the early-stage treatment model) with a similar method.

**VI. Consistency evaluation of features by Bland-Altman**

The Bland Altman (BA) method provides the bias information and 95% limits of agreement, and the latter demonstrate the range where 95% of all data points are expected to fall within. Quantitative comparisons using the BA indicated that the mean differences for all features were below1.380e-11 (absolute range: [6.322e-20, 1.380e-11]) and the standard deviations was 1.090. There were a total of 1551865 data points in YNCH, 74018 (4.8%) of which fall outside the 95% limits of agreement, which implied that about 95.2% of the data points fall within the 95% limits of agreement. Similar results were found at GPPH and SPCH dataset (Supplementary Table S2).

**Supplementary Figures**

**Figure S1**

**Figure S1.** Inclusion and exclusion criteria

**Figure S2**

**
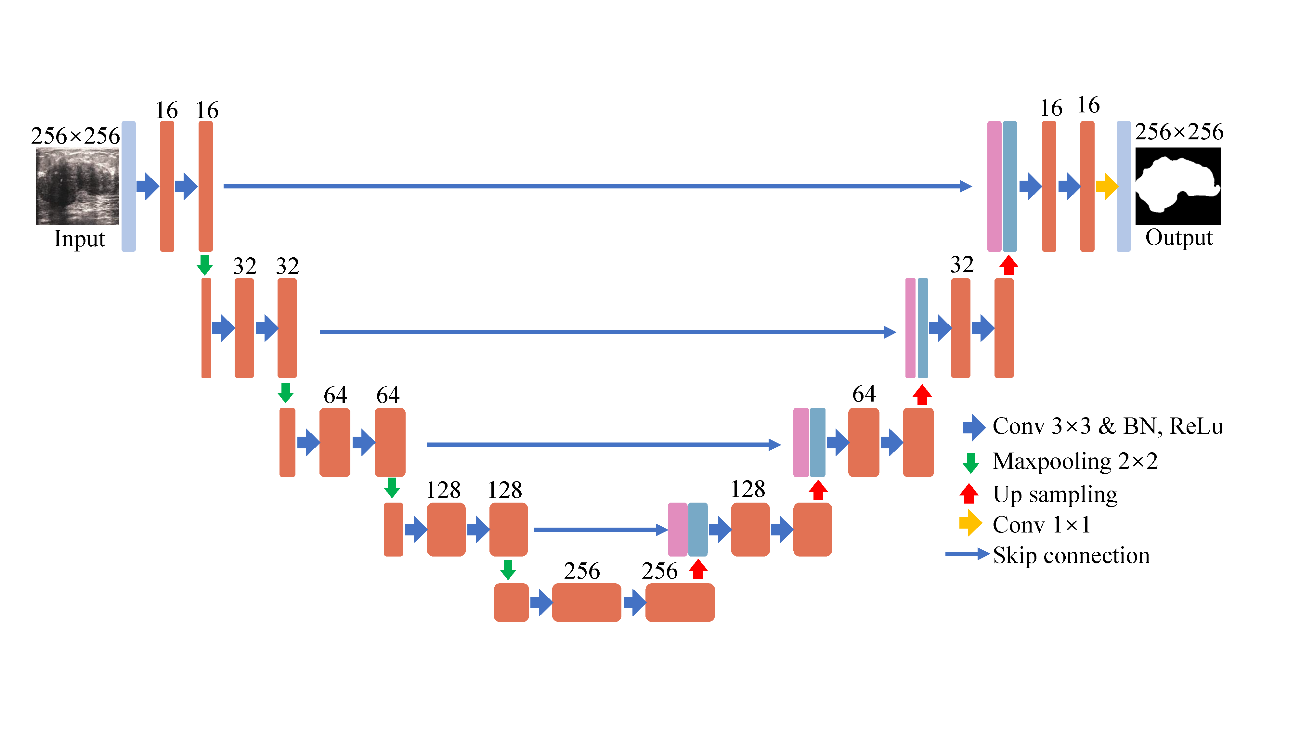
**

**Figure S2.** Detailed architecture of the U-Net.

**Figure S3**

**
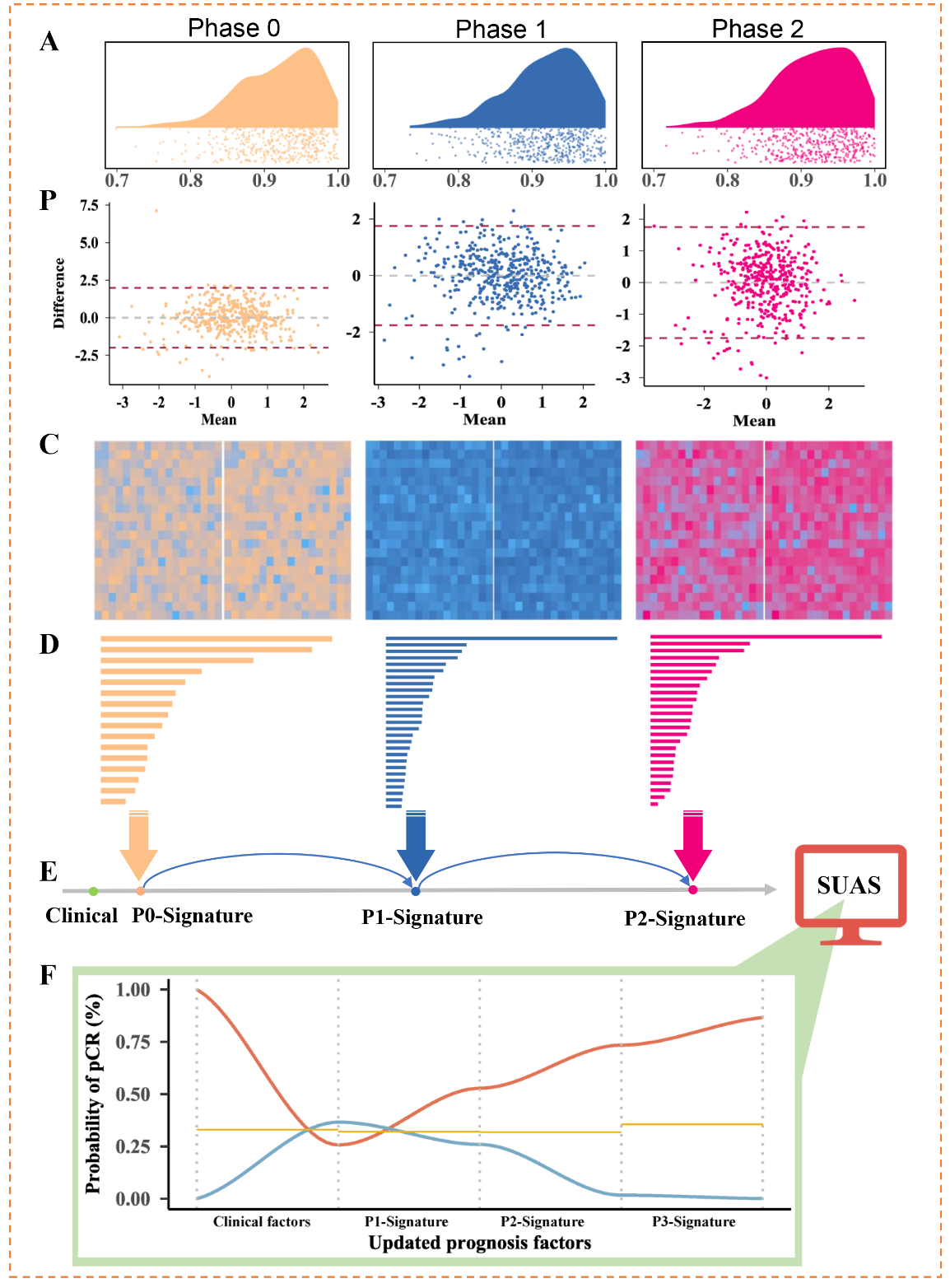
**

**Figure S3.** Flowchart of feature assessment and SUAS construction. Cosine similarity (A) and Bland-Altman test (B) were used to evaluate the feature similarity and consistency between manual segmentation and automated segmentation. SVD-R (C) and XGBoost algorithm (D) were used to reduce the dimension of the highly consistent features of automated segmentation. After further feature selection, the key features were used to construct the signatures, and SUAS was constructed based on the integrated signatures and clinical factors (E, F). SUAS: serial ultrasonography assessment system; SVD-R: singular value decomposition and reconstruction.

**Figure S4**

**Figure S4.** Principal component analysis plot and linear model for evaluating batch effect correction in three institutions. A), D) and G): before batch effect correction in YNCH, GDPH and SPCH institution respectively; B), E) and H): After batch effect correction using Combat method; C), F) and I): The proportion of radiomics features affected by the batch factor (machine) before and after batch correction.

**Figure S5**


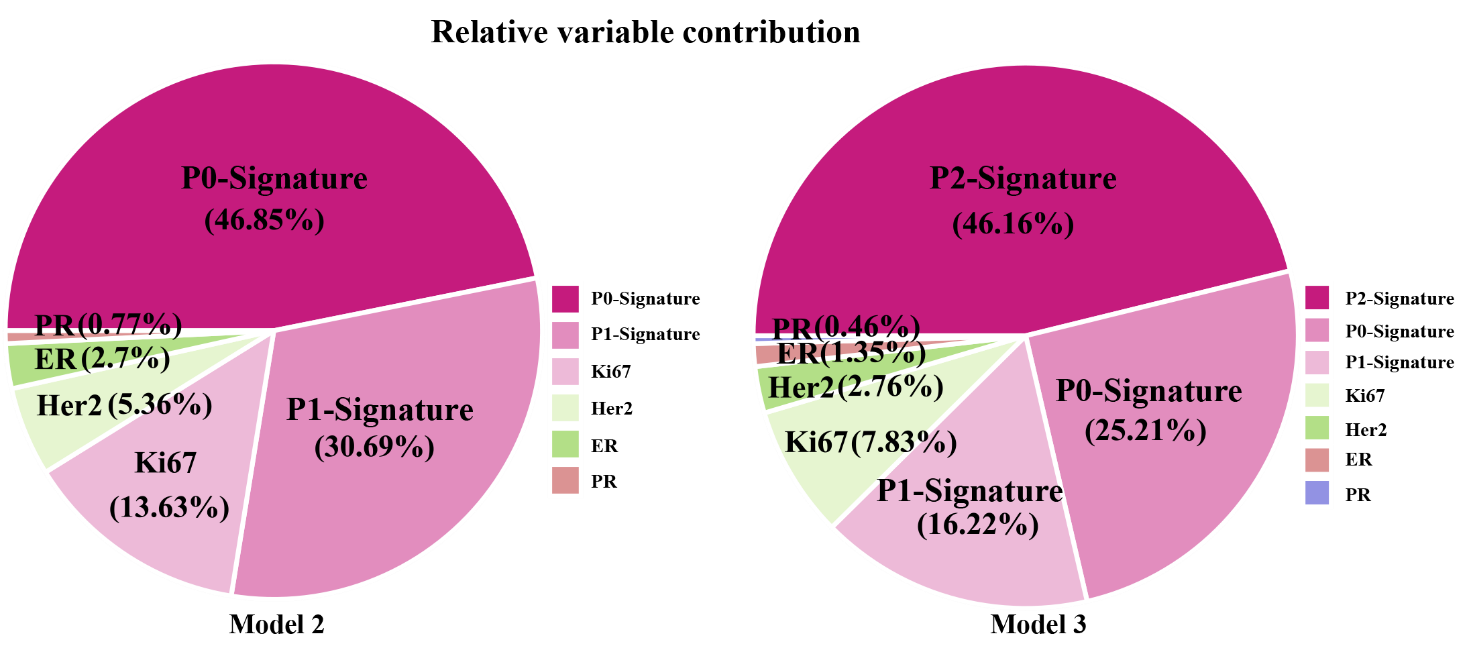


**Figure S5.** Relative variable contribution in Model 2 and Model 3. The relative importance of each prognostic factor to predict the pCR status in early-stage treatment (Model 2, left) and post-treatment (Model 3, right). *Abbreviations*: ER: estrogen receptor; PR: progesterone receptor; HER2: human epidermal growth factor receptor 2; P1: Phase 1 (early-stage treatment, namely, during the 1^st^-2^nd^ cycle of the neoadjuvant therapy); P2: Phase 2 (post-treatment).

**Figure S6**


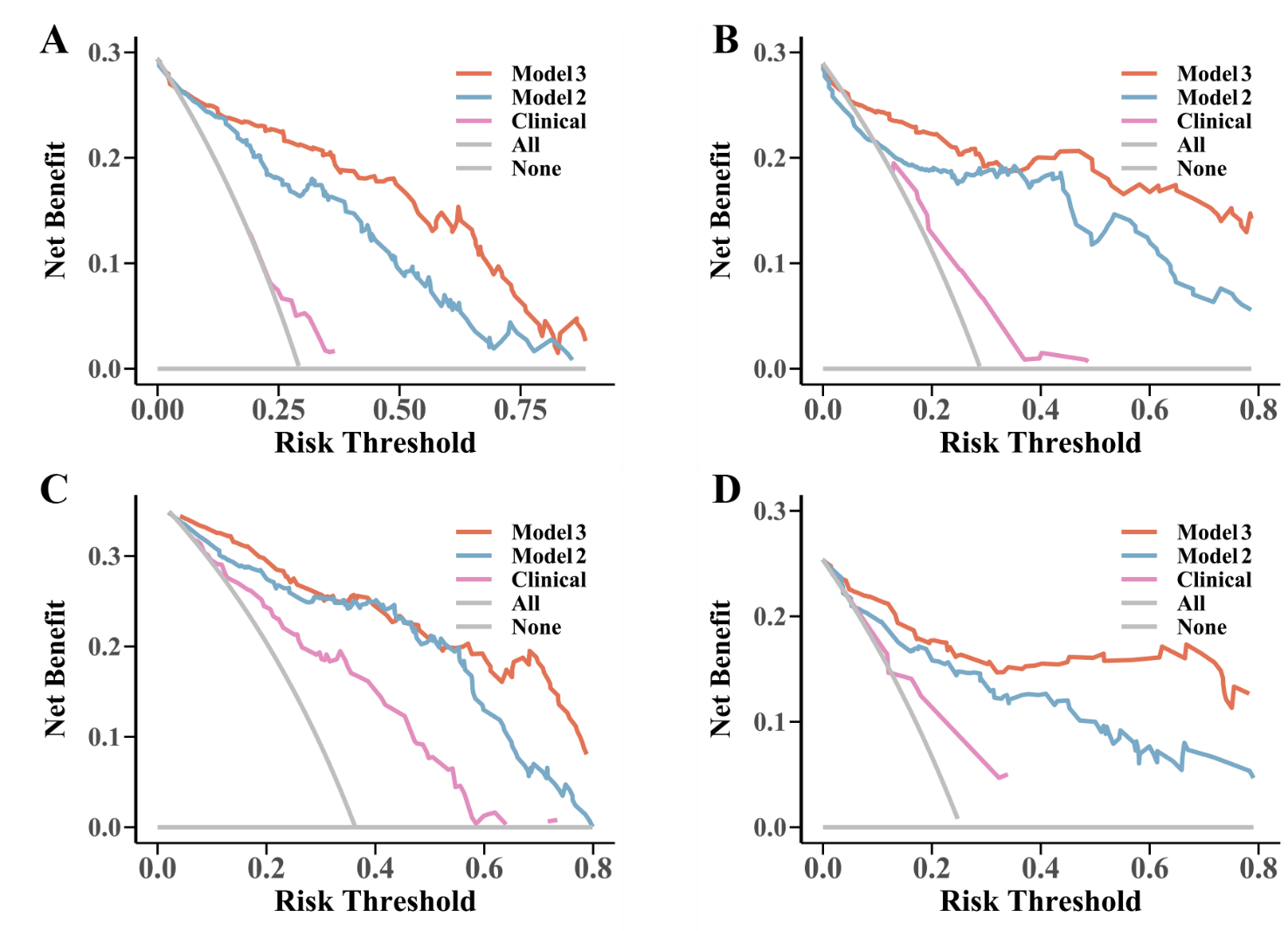


**Figure S6.** Clinical usefulness evaluation of Model 2, Model 3 and the clinicopathological model. The decision curve showed that when the risk threshold was greater than 0.1, more patients could benefit from Models 2-3 than the clinicopathological model in A) the training cohort, B) the internal validation cohort, C) the external validation cohort 1 (Guangdong Provincial People’s Hospital) and D) the external validation cohort 2 (Shanxi Cancer Hospital).

**Figure S7**


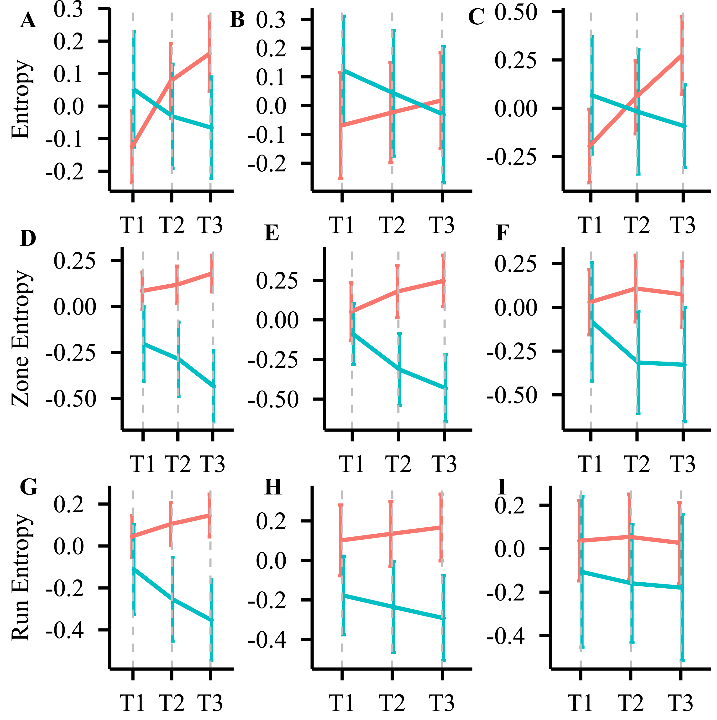


**Figure S7.** The changing trend of entropy-related features in three institutions. A), D) and G): YNCH institution; B), E) and H): GDPH institution; C), F) and I): SPCH institution. T1: pretreatment; T2: early-stage treatment; T3: posttreatment. red line: NpCR patients; blue line: pCR patients.

**Table S1. The ultrasonographic images acquisition parameters of the multiple centers**

| Hospital | Scanner | Probe | Frequency  (MHZ) | Machine index  (MI) | Thermal index of soft tissue (TIS) | Patients (No.) | | |
| --- | --- | --- | --- | --- | --- | --- | --- | --- |
|  |  |  |  |  |  | pCR | Non-pCR | *P*-value |
| Yunnan Cancer Hospital | Siemens (S2000) | 14L5 | 15 | 1·0 | 0·1 | 19 | 43 | 0.203 |
|  | Hitachi (Ascendus) | EUP-L53L | 9 | 1·0 | 0·4 | 126 | 268 |  |
|  | GE (LOGIQ E9) | ML6-159 | 14 | 0·3 | 0·1 | 59 | 149 |  |
|  | Philips (EPIQ7) | L12-5 | 12 | 0·6 | 0·1 | 29 | 99 |  |
|  | Mindray (DC-8) | L12-3E | 10 | 0·9 | 0·4 | 49 | 133 |  |
|  | TOSHIBA (Aplio 500) | PLT-1204BT | 10 | 1·0 | 0·1 | 47 | 136 |  |
|  | Others | - | - | - | - | 55 | 105 |  |
| Guangdong Provincial People's Hospital | Mindray (DC-8) | L12-3E | 10 | 0·9 | 0·4 | 4 | 20 | 0.054 |
|  | Hitachi (Ascendus) | EUP-L53L | 9 | 1·0 | 0·4 | 150 | 232 |  |
|  | TOSHIBA (Aplio 500) | PLT-1204BT | 10 | 1·0 | 0·1 | 71 | 151 |  |
|  | Others | - | - | - | - | 2 | 6 |  |
| Shanxi Cancer Hospital | Philips (HD15) | L12-5 | 12 | 1·0 | 0·4 | 22 | 69 | 0.617 |
|  | TOSHIBA (Aplio 500) | PLT-1204BT | 10 | 0·9 | 0·3 | 8 | 34 |  |
|  | GE (LOGIQ E9) | ML6-159 | 13 | 0·3 | 0·1 | 28 | 74 |  |
|  | Esaote (MyLab Twice) | LA523 | 10 | 1·0 | 0·4 | 40 | 105 |  |
|  | Hitachi (Prosound F37) | UST5412 | 10 | 1·0 | 0·4 | 13 | 34 |  |
|  | Others | - | - | - | - | 3 | 20 |  |

**Table S2. Similarity and consistency evaluation between automated segmentation and manual segmentation**

| Methods | YNCH | GPPH | SPCH |
| --- | --- | --- | --- |
| Cosine similarity | 0.918 [0.700-1.00] | 0.916 [0.734-1.00] | 0.915 [0.717-1.00] |
| Bland-Altman (Mean difference) | 1.380e-11 (95%CI: -2.706-2.706) | 1.437e-11 (95%CI: -2.752-2.752) | 5.525e-11 (95%CI: -2.666-2.666) |

Note: Cosine similarity were presented as mean (range [minimum- maximum]), Bland-Altman were presented as mean (95% CI). *Abbreviations*: 95% CI: 95% confidence interval; YNCH: Yunnan Cancer Hospital; GPPH: Guangdong Provincial People’s Hospital; SPCH: Shanxi Cancer Hospital.

**Table S3. Key features selected for the construction of phasales signatures based on ultrasound images at each single time point ultrasound images**

| **Phase features (N)** | **Feature names** | **Normalized Values**  **(mean ± standard deviation)** | |  |
| --- | --- | --- | --- | --- |
|  |  | **pCR (N=77)** | **NpCR (N=135)** | ***P*-value** |
| **P0 features**  **(N=12)** | db4.LL_glcm_Imc2 | -0.103±0.836 | 0.001±1.086 | 0.500 |
|  | db9.HH_glszm_ZoneEntropy | -0.201±0.946 | 0.115±1.015 | 0.039 |
|  | bior2.8.HH_glcm_ClusterShade | 0.179±0.858 | -0.102±1.062 | 0.013 |
|  | bior3.7.LH_firstorder_Skewness | -0.05±0.950 | 0.029±1.030 | 0.020 |
|  | rbio3.3.LH_gldm_GrayLevelVariance | -0.121±1.027 | 0.069±0.981 | 0.061 |
|  | db9.HL_glrlm_LongRunLowGrayLevelEmphasis | 0.138±1.043 | -0.079±0.970 | 0.025 |
|  | bior3.7.HL_glrlm_LongRunLowGrayLevelEmphasis | 0.217±1.116 | -0.124±0.909 | 0.019 |
|  | bior2.8.LL_glszm_SmallAreaLowGrayLevelEmphasis | 0.236 ±0.903 | -0.134 ±1.031 | <0.001 |
|  | sym8.LL_gldm_LargeDependenceLowGrayLevelEmphasis | 0.012 ±0.680 | -0.007 ±1.145 | 0.140 |
|  | sym3.LL_gldm_LargeDependenceLowGrayLevelEmphasis | 0.020 ±0.706 | -0.011 ±1.137 | 0.400 |
|  | rbio6.8.LL_gldm_LargeDependenceLowGrayLevelEmphasis | 0.005 ±0.668 | -0.003 ±1.149 | 0.200 |
|  | bior3.7.HL_gldm_SmallDependenceHighGrayLevelEmphasis | -0.157 ±0.764 | 0.089 ±1.105 | 0.007 |
| **P1 features**  **(N=11)** | bior3.7.LH_glcm_Idmn | -0.241 ±0.948 | 0.138 ±1.006 | 0.007 |
|  | sym3.LL_glszm_ZoneVariance | -0.092 ±0.758 | 0.052 ±1.114 | 0.004 |
|  | bior3.7.LL_glrlm_RunVariance | -0.071 ±0.865 | 0.040 ±1.071 | 0.005 |
|  | bior3.7.LH_glcm_JointEnergy | -0.178 ±1.007 | 0.101 ±0.985 | 0.070 |
|  | rbio3.3.HL_glrlm_RunEntropy | -0.206 ±0.921 | 0.117 ±1.027 | 0.013 |
|  | rbio3.3.LH_glszm_ZoneVariance | -0.073 ±0.730 | 0.042 ±1.126 | 0.003 |
|  | coif5.LL_glszm_LargeAreaEmphasis | -0.099 ±0.727 | 0.056 ±1.125 | 0.003 |
|  | original_glszm_LargeAreaHighGrayLevelEmphasis | -0.079 ±0.553 | 0.045 ±1.181 | 0.002 |
|  | bior2.8.LH_glszm_LargeAreaHighGrayLevelEmphasis | 0.008 ±1.108 | -0.004 ±0.937 | 0.003 |
|  | sym3.HH_glszm_GrayLevelNonUniformityNormalized | 0.173 ±0.929 | -0.098 ±1.029 | 0.046 |
|  | bior3.7.HL_glszm_GrayLevelNonUniformityNormalized | 0.177 ±0.907 | -0.101 ±1.039 | 0.060 |
| **P2 features**  **(N=9)** | coif5.LH_glcm_Idmn | -0.329 ±1.015 | 0.188 ±0.944 | <0.001 |
|  | db9.LH_firstorder_Median | -0.055 ±1.335 | 0.031 ±0.749 | 0.6 |
|  | original_glszm_ZoneEntropy | -0.432 ±0.933 | 0.246 ±0.956 | <0.001 |
|  | rbio6.8.LH_glrlm_RunEntropy | -0.173 ±1.008 | 0.099 ±0.986 | 0.054 |
|  | bior3.7.LL_glcm_MaximumProbability | -0.148 ±0.926 | 0.085 ±1.034 | 0.11 |
|  | coif5.LH_glszm_LowGrayLevelZoneEmphasis | 0.202 ±1.109 | -0.115 ±0.917 | 0.012 |
|  | db4.LH_glszm_LargeAreaLowGrayLevelEmphasis | -0.111 ±0.537 | 0.063 ±1.183 | 0.010 |
|  | coif5.LH_glszm_LargeAreaLowGrayLevelEmphasis | -0.157 ±0.561 | 0.090 ±1.172 | 0.011 |
|  | rbio3.3.LL_gldm_DependenceNonUniformityNormalized | 0.043 ±0.914 | -0.024 ±1.048 | 0.4 |

Note: LL, LH, HH and HL represents wavelet transform directions. *Abbreviations*: glcm: gray level cooccurrence matrix; glszm: gray level size zone matrix; glrlm: gray level run length matrix; gldm: gray level dependence matrix; P0: Phase 0 (pretreatment); P1: Phase 1 (early-stage treatment, namely, during the 1^st^-2^nd^ cycle of the neoadjuvant therapy); P2: Phase 2 (post-treatment); pCR: pathological complete response; NpCR: non pathological complete response.

**Table S4. Multivariate analysis of clinicopathological model, Model 2 (the early-treatment model) and Model 3 (the post-treatment model)**

|  | **Clinicopathological model** | |  | **Model 2** | |  | **Model 3** | |  |
| --- | --- | --- | --- | --- | --- | --- | --- | --- | --- |
|  | **OR** | **95%CI** | **p-value** | **OR** | **95%CI** | **p-value** | **OR** | **95%CI** | **p-value** |
| **Ki67** |  |  |  |  |  |  |  |  |  |
| Negative | reference |  |  |  |  |  |  |  |  |
| Positive | 1.436 | 0.783-2.633 | 0.240 | 1.716 | 0.815-3.615 | 0.153 | 1.985 | 0.858-4.594 | 0.107 |
| **ER** |  |  |  |  |  |  |  |  |  |
| Negative | reference |  |  |  |  |  |  |  |  |
| Positive | 0.742 | 0.333-1.657 | 0.465 | 0.559 | 0.212-1.470 | 0.236 | 0.700 | 0.242-2.024 | 0.509 |
| **PR** |  |  |  |  |  |  |  |  |  |
| Negative | reference |  |  |  |  |  |  |  |  |
| Positive | 0.990 | 0.427-2.302 | 0.984 | 0.701 | 0.258-1.904 | 0.484 | 0.768 | 0.231-2.548 | 0.664 |
| **Her2** |  |  |  |  |  |  |  |  |  |
| Negative | reference |  |  |  |  |  |  |  |  |
| Positive | 0.937 | 0.078-11.320 | 0.959 | 1.062 | 0.369-3.055 | 0.911 | 1.036 | 0.063-16.92 | 0.980 |
| **Subtype** |  |  |  |  |  |  |  |  |  |
| Triple-negative | reference |  |  |  |  |  |  |  |  |
| HER2(+) | 2.047 | 0.161-25.97 | 0.578 | 1.375 | 0.083-22.90 | 0.823 | 1.271 | 0.075-21.44 | 0.867 |
| HER2(-)&HR(+) | 1.194 | 0.361-3.945 | 0.770 | 1.275 | 0.305-5.331 | 0.738 | 1.378 | 0.272-6.987 | 0.697 |
| **P0-Signature** | — | — | — | 2.357 | 1.531-3.629 | <0.001 | 1.881 | 1.222-2.894 | 0.004 |
| **P1-Signature** | — | — | — | 2.455 | 1.721-3.502 | <0.001 | 2.094 | 1.468-2.987 | <0.001 |
| **P2-Signature** | — | — | — | — | — | — | 2.097 | 1.543-2.849 | <0.001 |

Note: Model 2 was built based on the P0-Signature plus the P1-Signature. And Model 3 was based on Model 2 plus the P2-Signature. Abbreviations: ER: estrogen receptor; PR: progesterone receptor; HER2: human epidermal growth factor receptor; P0: Phase 0 (pretreatment); P1: Phase 1 (early-stage treatment, namely, during the 1st-2nd cycle of the neoadjuvant therapy); P2: Phase 2 (post-treatment); OR: odds ratio; 95%CI: 95% confidence interval.

**Table S5 Prediction performance of the signatures**

|  | **Training cohort** | **Internal validation cohort** | **External test cohort 1** | **External test cohort 2** |
| --- | --- | --- | --- | --- |
| **Radiologist interpretation** | 0.547 (0.498-0.595) | 0.536 (0.483-0.589) | 0.632 (0.570-0.693) | 0.724 (0.644-0.804) |
| **Clinicopathological model** | 0.609 (0.531-0.686) | 0.651 (0.570-0.732) | 0.724 (0.656-0.793) | 0.599 (0.497-0.701) |
| **P0-Signature** |  |  |  |  |
| AUC (95% CI) | 0.800 (0.742-0.859) | 0.821 (0.750-0.892) | 0.810 (0.748-0.873) | 0.812 (0.718-0.906) |
| Sensitivity (95% CI) | 0.915 (0.851-0.980) | 0.772 (0.663-0.881) | 0.818 (0.732-0.904) | 0.816 (0.693-0.939) |
| Specificity (95% CI) | 0.620 (0.547-0.693) | 0.857(0.799-0.915) | 0.815 (0.749-0.880) | 0.866 (0.803-0.929) |
| Accuracy (95% CI) | 0.707 (0.705-0.708) | 0.832 (0.831-0.834) | 0.816 (0.815-0.817) | 0.853 (0.852-0.855) |
| PPV (95% CI) | 0.500 (0.414-0.586) | 0.688 (0.574-0.801) | 0.716 (0.622-0.810) | 0.674 (0.538-0.809) |
| NPV (95% CI) | 0.964 (0.905-0.988) | 0.902 (0.852-0.953) | 0.887 (0.831-0.943) | 0.933 (0.885-0.981) |
| **P1-Signature** |  |  |  |  |
| AUC (95% CI) | 0.837 (0.781-0.893) | 0.851 (0.782-0.919) | 0.834 (0.779-0.889) | 0.829 (0.756-0.902) |
| Sensitivity (95% CI) | 0.761 (0.661-0.860) | 0.825 (0.726-0.923)- | 0.961 (0.918-1.00) | 0.842 (0.726-0.958) |
| Specificity (95% CI) | 0.836 (0.781-0.892) | 0.864 (0.808-0.921) | 0.689 (0.611-0.767) | 0.741 (0.660-0.822) |
| Accuracy (95% CI) | 0.814 (0.813-0.815) | 0.853 (0.852-0.854) | 0.788 (0.786-0.789) | 0.767 (0.764-0.769) |
| PPV (95% CI) | 0.659 (0.556-0.761) | 0.712 (0.603-0.821) | 0.638 (0.550-0.725) | 0.525 (0.399-0.650) |
| NPV (95% CI) | 0.894 (0.846-0.941) | 0.924 (0.878-0.969) | 0.969 (0.934-1.00) | 0.933 (0.880-0.985) |
| **P2-Signature** |  |  |  |  |
| AUC (95% CI) | 0.864 (0.815-0.912) | 0.882 (0.828-0.935) | 0.862 (0.813-0.912) | 0.851 (0.777-0.926) |
| Sensitivity (95% CI) | 0.817 (0.727-0.907) | 0.807 (0.705-0.909) | 0.753 (0.657-0.850) | 0.763 (0.628-0.898) |
| Specificity (95% CI) | 0.772 (0.709-0.835) | 0.879 (0.824-0.933) | 0.889 (0.836-0.942) | 0.929 (0.881-0.976) |
| Accuracy (95% CI) | 0.785 (0.784-0.786) | 0.858 (0.857-0.807) | 0.840 (0.838-0.841) | 0.887 (0.885-0.888) |
| PPV (95% CI) | 0.598(0.500-0.696) | 0.730 (0.621-0.840) | 0.795 (0.702-0.887) | 0.784 (0.651-0.916) |
| NPV (95% CI) | 0.910 (0.864-0.957) | 0.918 (0.871-0.964) | 0.863 (0.806-0.920) | 0.920 (0.870-0.970) |

*Abbreviations*: AUC: area under receiver operating characteristic curve; PPV: positive predictive values; NPV: negative predictive values; P0: Phase 0 (pretreatment); P1: Phase 1 (early-stage treatment, namely, during the 1^st^-2^nd^ cycle of the neoadjuvant therapy); P2: Phase 2 (post-treatment).

**Table S6. AUCs of early-pretreatment model (Model 2) and post-treatment model (Model 3) in breast cancer subtypes**

| Model | Subtype | Training cohort | Internal  validation cohort | External  test cohort 1 | External  test cohort 2 |
| --- | --- | --- | --- | --- | --- |
| Model 2 |  |  |  |  |  |
|  | HER2+ (95% CI) | 0.901 (0.843-0.960) | 0.856 (0.736-0.976) | 0.912 (0.857-0.968) | 0.872 (0.746-0.998) |
|  | Triple Negative (95% CI) | 0.812 (0.600-1.00) | 0.931 (0.827-1.00) | 0.798 (0.629-0.967) | 0.840 (0.659-1.00) |
|  | HR+, HER2- (95% CI) | 0.857 (0.790-0.925) | 0.889 (0.790-0.988) | 0.903 (0.788-1.00) | 0.868 (0.697-1.00) |
| Model 3 |  |  |  |  |  |
|  | HER2+ (95% CI) | 0.955 (0.915-0.996) | 0.876 (0.763-0.989) | 0.945 (0.902-0.987) | 0.919 (0.823-1.00) |
|  | Triple Negative (95% CI) | 0.932 (0.821-1.00) | 0.922 (0.811-1.00) | 0.802 (0.635-0.968) | 0.853 (0.684-1.00) |
|  | HR+, HER2- (95% CI) | 0.899 (0.836-0.962) | 0.945 (0.879-1.00) | 0.939 (0.853-1.00) | 0.930 (0.834-1.00) |

Note: Model 2 was based on the Phase 0 (pretreatment) signature plus the Phase 1 (early-stage treatment, namely, during the 1^st^-2^nd^ cycle of the neoadjuvant therapy) signature. And Model 3 was based on Model 2 plus the Phase 2 (post-treatment) signature. *Abbreviations:* AUC: area under receiver operating characteristic curve; HR: hormone receptor; HER2: human epidermal growth factor receptor 2; 95%CI: 95% confidence interval; Internal validation: the internal validation cohort;

**Table S7. NRI test for prediction improvements of SUAS compared to single-time signature in multiple cohorts**

| **Model** | **Training cohort** | |  | **Internal**  **validation cohort** | |  | **External**  **test cohort 1** | |  | **External**  **test cohort 2** | |
| --- | --- | --- | --- | --- | --- | --- | --- | --- | --- | --- | --- |
|  | **NRI (95% CI)** | ***P-*value** |  | **NRI (95% CI)** | ***P-*value** |  | **NRI (95% CI)** | ***P-*value** |  | **NRI (95% CI)** | ***P-*value** |
| SUAS | — | — |  | — | — |  | — | — |  | — | — |
| P0-Signatue | 1.250 (1.045-1.456) | <0.0001 |  | 1.075 (0.850-1.300) | <0.0001 |  | 0.803 (0.582-1.025)) | <0.0001 |  | 0.995 (0.729-1.261) | <0.0001 |
| P1-Signatue | 1.064 (0.846-1.282) | <0.0001 |  | 1.168 (0.947-1.389) | <0.0001 |  | 1.088 (0.865-1.312) | <0.0001 |  | 0.926 (0.623-1.229) | <0.0001 |
| P2-Signatue | 0.855 (0.627-1.083) | <0.0001 |  | 1.063 (0.815-1.312) | <0.0001 |  | 1.122 (0.893-0.135) | <0.0001 |  | 0.970 (0.678-0.126) | <0.0001 |

Note: SUAS: serial ultrasonography assessment system, integrated the pre-, early-stage and post-treatment signatures.

Abbreviations: NRI: net reclassification index; 95% CI: 95% confidence interval; P0: Phase 0 (pretreatment); P1: Phase 1 (early-stage treatment, namely, during the 1st-2nd cycle of the neoadjuvant therapy); P2: Phase 2 (post-treatment).

**Table S8. IDI test for prediction improvements of SUAS compared to single-time signature in multiple cohorts**

| **Model** | **Training cohort** | |  | **Internal**  **validation cohort** | |  | **External**  **test cohort 1** | |  | **External**  **test cohort 2** | |
| --- | --- | --- | --- | --- | --- | --- | --- | --- | --- | --- | --- |
|  | **IDI (95% CI)** | ***P-*value** |  | **NRI (95% CI)** | ***P-*value** |  | **NRI (95% CI)** | ***P-*value** |  | **NRI (95% CI)** | ***P-*value** |
| SUAS | — | — |  | — | — |  | — | — |  | — | — |
| P0-Signatue | 0.359 (0.297-0.421) | <0.0001 |  | 0.369 (0.287-0.451) | <0.0001 |  | 0.357 (0.258-0.456) | <0.0001 |  | 0.305 (0.226-0.384) | <0.0001 |
| P1-Signatue | 0.263 (0.207-0.318) | <0.0001 |  | 0.268 (0.196-0.340) | <0.0001 |  | 0.283 (0.216-0.349) | <0.0001 |  | 0.285 (0.166-0.405) | <0.0001 |
| P2-Signatue | 0.183 (0.136-0.230) | <0.0001 |  | 0.181 (0.103-0.260) | <0.0001 |  | 0.179 (0.111-0.246) | <0.0001 |  | 0.242 (0.124-0.359) | <0.0001 |

Note: SUAS: serial ultrasonography assessment system, integrated the pre-, early-stage and post-treatment signatures.

Abbreviations: IDI: integrated discrimination improvement; 95% CI: 95% confidence interval; P0: Phase 0 (pretreatment); P1: Phase 1 (early-stage treatment, namely, during the 1st-2nd cycle of the neoadjuvant therapy); P2: Phase 2 (post-treatment).

**Table S9. Prediction performance of the signatures and SUAS in randomly split datasets.**

|  | **Training cohort** | **Validation cohort** | **Test cohort** |
| --- | --- | --- | --- |
| **P0-Signature (Model 1)** |  |  |  |
| AUC (95% CI) | 0.780 (0.734-0.827) | 0.756 (0.676-0.836) | 0.760 (0.674-0.847) |
| Sensitivity (95% CI) | 0.669 (0.593-0.745) | 0.795 (0.676-0.915) | 0.608 (0.474-0.742) |
| Specificity (95% CI) | 0.808 (0.765-0.850) | 0.672 (0.587-0.758) | 0.853 (0.787-0.920) |
| Accuracy (95% CI) | 0.765 (0.764-0.766) | 0.706 (0.704-0.709) | 0.775 (0.773-0.777) |
| PPV (95% CI) | 0.607 (0.532-0.682) | 0.479 (0.365-0.594) | 0.660 (0.524-0.795) |
| NPV (95% CI) | 0.846 (0.806-0.886) | 0.897 (0.833-0.961) | 0.823 (0.753-0.893) |
| **P1-Signature** |  |  |  |
| AUC (95% CI) | 0.840 (0.800-0.879) | 0.846 (0.780-0.912) | 0.852 (0.786-0.919) |
| Sensitivity (95% CI) | 0.791 (0.725-0.856) | 0.727 (0.596-0.859) | 0.824 (0.719-0.928) |
| Specificity (95% CI) | 0.757 (0.711-0.803) | 0.836 (0.769-0.904) | 0.780 (0.702-0.858) |
| Accuracy (95% CI) | 0.767 (0.766-0.768) | 0.806 (0.804-0.808) | 0.794 (0.792-0.796) |
| PPV (95% CI) | 0.591 (0.522-0.659) | 0.627 (0.495-0.768) | 0.636 (0.520-0.752) |
| NPV (95% CI) | 0.890 (0.854-0.927) | 0.890 (0.831-0.949) | 0.904 (0.845-0.964) |
| **P2-Signature** |  |  |  |
| AUC (95% CI) | 0.842 (0.805-0.879) | 0.867 (0.806-0.928) | 0.850 (0.790-0.911) |
| Sensitivity (95% CI) | 0.757 (0.688-0.826) | 0.773 (0.649-0.897) | 0.922 (0.848-0.995) |
| Specificity (95% CI) | 0.790 (0.746-0.834) | 0.845 (0.779-0.911) | 0.679 (0.591-0.767) |
| Accuracy (95% CI) | 0.780 (0.779-0.780) | 0.825 (0.823-0.827) | 0.756 (0.754-0.759) |
| PPV (95% CI) | 0.615 (0.545-0.686) | 0.654 (0.525-0.783) | 0.573 (0.466-0.680) |
| NPV (95% CI) | 0.880 (0.843-0.916) | 0.907 (0.853-0.962) | 0.949 (0.900-0.998) |
| **Model 2** |  |  |  |
| AUC (95% CI) | 0.877 (0.842-0.913) | 0.869 (0.808-0.931) | 0.883 (0.822-0.944) |
| Sensitivity (95% CI) | 0.777 (0.710-0.844) | 0.705 (0.570-0.839) | 0.804 (0.695-0.913) |
| Specificity (95% CI) | 0.868 (0.831-0.904) | 0.922 (0.874-0.971) | 0.862 (0.798-0.927) |
| Accuracy (95% CI) | 0.840 (0.839-0.840) | 0.863 (0.861-0.864) | 0.844 (0.842-0.845) |
| PPV (95% CI) | 0.723 (0.654-0.793) | 0.775 (0.646-0.904) | 0.732 (0.616-0.848) |
| NPV (95% CI) | 0.898 (0.864-0.931) | 0.892 (0.836-0.947) | 0.904 (0.847-0.961) |
| **Model 3** |  |  |  |
| AUC (95% CI) | 0.919 (0.891-0.946) | 0.928 (0.885-0.970) | 0.912 (0.866-0.958) |
| Sensitivity (95% CI) | 0.878 (0.826-0.931) | 0.864 (0.762-0.965) | 0.725 (0.603-0.848) |
| Specificity (95% CI) | 0.856 (0.818-0.894) | 0.914 (0.863-0.965) | 0.945 (0.902-0.988) |
| Accuracy (95% CI) | 0.863 (0.862-0.863) | 0.900 (0.899-0.901) | 0.875 (0.874-0.876) |
| PPV (95% CI) | 0.730 (0.665-0.796) | 0.792 (0.677-0.907) | 0.860 (0.757-0.964) |
| NPV (95% CI) | 0.941 (0.914-0.967) | 0.946 (0.905-0.988) | 0.880 (0.822-0.939) |

Note. Model 1 was built based on the Phase 0 (pretreatment) signature (P0-Siganture). Model 2 was based on Model 1 plus the Phase 1 (early-stage treatment, namely, during the 1st-2nd cycle of the neoadjuvant therapy) signature (P1-Signature). And Model 3 was based on Model 2 plus the Phase 2 (post-treatment) signature (P2-Signature). Abbreviations: AUC: area under receiver operating characteristic curve; PPV: positive predictive values; NPV: negative predictive values.

**References**

1. Ronneberger O, Fischer P, Brox T. U-net: Convolutional networks for biomedical image segmentation. International Conference on Medical image computing and computer-assisted intervention; 2015: Springer; 2015. p. 234-41.

2. van Griethuysen JJM, Fedorov A, Parmar C, et al. Computational Radiomics System to Decode the Radiographic Phenotype. *Cancer Res* 2017; **77**(21): e104-e7.

3. Johnson WE, Li C, Rabinovic A. Adjusting batch effects in microarray expression data using empirical Bayes methods. *Biostatistics (Oxford, England)* 2007; **8**(1): 118-27.

4. Fortin JP, Cullen N, Sheline YI, et al. Harmonization of cortical thickness measurements across scanners and sites. *Neuroimage* 2018; **167**: 104-20.

5. Fortin J-P, Parker D, Tunç B, et al. Harmonization of multi-site diffusion tensor imaging data. *Neuroimage* 2017; **161**: 149-70.

6. Chen T, Guestrin CJNY, NY, USA: ACM. XGBoost: A scalable tree boosting system In Proceedings of the 22Nd ACM SIGKDD International Conference on Knowledge Discovery and Data Mining,(pp. 785–794). 2016; **10**(2939672.2939785).

7. Guyon I, Weston J, Barnhill S, Vapnik V. Gene Selection for Cancer Classification using Support Vector Machines. *Machine Learning* 2002; **46**(1): 389-422.
